# Supplementary material for: LAST, a c-Myc-inducible long noncoding RNA, cooperates with CNBP to promote CCND1 mRNA stability in human cells
Source: eLife. 2017 Dec 4;6:e30433. doi: 10.7554/eLife.30433 (PMC5739540; doi:10.7554/eLife.30433)
Supplement: Supplementary file 1. — Information of the selected lncRNAs is colored. [file elife-30433-supp1.doc]

**Supplementary file 1. LncRNA expression microarray data**

| **Probe Name** | **P-value** | **Absolute Fold change([D+] vs [D-])** | **Regulation([D+] vs [D-])** | **Type** | **Sequence Name** | **Gene Symbol** |
| --- | --- | --- | --- | --- | --- | --- |
| ASHGA5P030920 | 0.000194599 | 14.97566885 | down | noncoding | ENST00000563991 | RP11-359K18.3 |
| ASHGA5P027749 | 0.000116546 | 27.89343428 | down | noncoding | ENST00000513358 (lncRNA-51) | RP11-253E3.3 |
| ASHGA5P026593 | 8.18437E-06 | 10.07287073 | down | noncoding | ENST00000529369 (lncRNA-52) | RP11-660L16.2 |
| ASHGA5P044770 | 8.07319E-05 | 8.632201649 | down | noncoding | ENST00000563059 | KB-1836B5.1 |
| ASHGA5P038919 | 0.00117675 | 12.55389173 | down | noncoding | ENST00000569034 | RP11-15N24.4 |
| ASHGA5P043828 | 0.000109571 | 27.24986794 | down | noncoding | ENST00000419422 | RP11-132A1.4 |
| ASHGA5P035590 | 5.5636E-05 | 22.44101357 | down | noncoding | ENST00000568928 | RP11-16P6.1 |
| ASHGA5P038317 | 2.80311E-05 | 21.36905346 | down | noncoding | ENST00000497543 | FAM86DP |
| ASHGA5P050387 | 5.74283E-05 | 16.21130171 | down | noncoding | ENST00000547349 | RP11-320M2.1 |
| ASHGA5P042574 | 0.00221943 | 16.09279042 | down | noncoding | ENST00000440395 | TRAF3IP2-AS1 |
| ASHGA5P037905 | 1.37617E-05 | 15.14070838 | down | noncoding | ENST00000451486 | CTA-292E10.6 |
| ASHGA5P025613 | 9.79074E-05 | 15.08118827 | down | noncoding | NR_004389 | SNORA16B |
| ASHGA5P045955 | 0.001195908 | 15.03921334 | down | noncoding | AU185412R |  |
| ASHGA5P025619 | 5.39405E-06 | 14.64328874 | down | noncoding | NR_004399 | SNORD86 |
| ASHGA5P014668 | 1.38792E-08 | 14.45192132 | down | noncoding | ENST00000412301 | AC129778.2 |
| ASHGA5P045245 | 6.51443E-07 | 13.83509425 | down | noncoding | ENST00000452477 | RP11-292F9.2 |
| ASHGA5P051064 | 0.000126053 | 13.7077353 | down | noncoding | ENST00000447343 | RP4-583P15.10 |
| ASHGA5P031306 | 0.000129345 | 13.69279034 | down | noncoding | ENST00000432536 | GAS5 |
| ASHGA5P025478 | 0.000166638 | 13.58870466 | down | noncoding | NR_003073 | SNORD91B |
| ASHGA5P028281 | 0.000355488 | 13.52935916 | down | noncoding | ENST00000547021 | RP11-686G8.2 |
| ASHGA5P024850 | 0.000479148 | 13.35773321 | down | noncoding | hsa-mir-5087 | MI0017976 |
| ASHGA5P022230 | 0.000211722 | 13.17136597 | down | noncoding | ENST00000552933 | RP11-386G11.5 |
| ASHGA5P037041 | 0.001477608 | 13.05139941 | down | noncoding | ENST00000400436 | SNHG11 |
| ASHGA5P018194 | 5.6026E-05 | 13.05087171 | down | noncoding | ENST00000450469 | RP4-784A16.3 |
| ASHGA5P022874 | 3.56352E-06 | 13.04018863 | down | noncoding | NR_033249 | GCSH |
| ASHGA5P018301 | 0.002022012 | 12.68593016 | down | noncoding | uc002ixb.3 | BC017255 |
| ASHGA5P030091 | 0.000156905 | 12.47753143 | down | noncoding | ENST00000561191 | RP11-265M18.2 |
| ASHGA5P020318 | 1.05076E-05 | 12.4190364 | down | noncoding | ENST00000511488 | FAM86EP |
| ASHGA5P036937 | 6.6878E-05 | 12.40028465 | down | noncoding | TCONS_00025394 | XLOC_012209 |
| ASHGA5P032914 | 5.29911E-05 | 12.31645928 | down | noncoding | ENST00000584923 | SNORD3A |
| ASHGA5P020049 | 1.87914E-06 | 12.20280777 | down | noncoding | ENST00000507301 | FAM86EP |
| ASHGA5P016758 | 0.002574394 | 12.14426103 | down | noncoding | ENST00000433905 | RP4-583P15.10 |
| ASHGA5P024136 | 0.000235371 | 12.07375924 | down | noncoding | hsa-mir-19b-1 | MI0000074 |
| ASHGA5P031650 | 0.000101619 | 11.91973838 | down | noncoding | ENST00000450909 | CTD-2574D22.2 |
| ASHGA5P027005 | 4.65394E-05 | 11.65922239 | down | noncoding | ENST00000455744 | RP1-120G22.11 |
| ASHGA5P014887 | 2.95546E-07 | 11.5802752 | down | noncoding | ENST00000414386 | RP11-328M4.2 |
| ASHGA5P000097 | 2.57421E-06 | 11.48734375 | down | noncoding | BC030084 |  |
| ASHGA5P025145 | 5.14454E-05 | 11.45878652 | down | noncoding | hsa-mir-92a-1 | MI0000093 |
| ASHGA5P050141 | 0.002363195 | 11.40304195 | down | noncoding | TCONS_00014235 | XLOC_006301 |
| ASHGA5P030919 | 7.8402E-06 | 11.35103779 | down | noncoding | ENST00000570409 | RP11-461A8.4 |
| ASHGA5P047598 | 0.000372157 | 11.33277789 | down | noncoding | ENST00000432120 | RP11-119K6.6 |
| ASHGA5P014151 | 2.69036E-05 | 11.30353956 | down | noncoding | NR_027349 | MIR17HG |
| ASHGA5P050123 | 0.013122099 | 11.21920103 | down | noncoding | TCONS_00013644 | XLOC_006301 |
| ASHGA5P030064 | 2.36062E-05 | 11.03789886 | down | noncoding | ENST00000456382 | RP11-29H23.4 |
| ASHGA5P027514 | 0.000395867 | 11.02566041 | down | noncoding | NR_027157 | TMPO-AS1 |
| ASHGA5P025287 | 0.001164437 | 10.99361177 | down | noncoding | NR_001276 | SNORD56B |
| ASHGA5P049487 | 0.009899316 | 10.93452541 | down | noncoding | NR_033827 | KDM4A-AS1 |
| ASHGA5P043867 | 0.000195068 | 10.92310594 | down | noncoding | TCONS_00018595 | XLOC_008935 |
| ASHGA5P032010 | 7.10777E-06 | 10.87159466 | down | noncoding | NR_036480 | LOC100128881 |
| ASHGA5P057644 | 3.08954E-05 | 10.86282172 | down | noncoding | TCONS_00020322 | XLOC_009653 |
| ASHGA5P025477 | 0.000783164 | 10.77908839 | down | noncoding | NR_003072 | SNORD91A |
| ASHGA5P051881 | 2.39276E-05 | 10.76762774 | down | noncoding | ENST00000512092 | FAM86EP |
| ASHGA5P025616 | 0.001749768 | 10.69696287 | down | noncoding | NR_004396 | SNORD1B |
| ASHGA5P045145 | 5.51341E-05 | 10.68822615 | down | noncoding | ENST00000439876 | RP11-4E23.2 |
| ASHGA5P027966 | 0.000304401 | 10.59849591 | down | noncoding | ENST00000547395 | RP11-386G11.5 |
| ASHGA5P039023 | 1.45969E-05 | 10.49038582 | down | noncoding | ENST00000464612 | SNHG12 |
| ASHGA5P048488 | 0.000131182 | 10.46933837 | down | noncoding | ENST00000526453 | RP11-110I1.12 |
| ASHGA5P053696 | 1.71013E-05 | 10.46115204 | down | noncoding | uc022bhk.1 | FAM27E3 |
| ASHGA5P038060 | 2.425E-05 | 10.32908461 | down | noncoding | uc001cvn.1 | AK097571 |
| ASHGA5P025041 | 0.001586332 | 10.2424201 | down | noncoding | hsa-mir-621 | MI0003635 |
| ASHGA5P041507 | 0.003154206 | 10.22892999 | down | noncoding | ENST00000521204 | CTB-11I22.1 |
| ASHGA5P048257 | 0.000250011 | 10.10721697 | down | noncoding | ENST00000528089 | FAM86C2P |
| ASHGA5P057372 | 9.56429E-06 | 10.06668636 | down | noncoding | TCONS_00016406 | XLOC_007777 |
| ASHGA5P028039 | 2.38977E-05 | 9.969176249 | down | noncoding | NR_026657 | LOC100240734 |
| ASHGA5P040105 | 7.12308E-06 | 9.9010578 | down | noncoding | uc003ifn.3 | AK090904 |
| ASHGA5P043247 | 0.003876096 | 9.850456375 | down | noncoding | ENST00000414948 | ENO1-IT1 |
| ASHGA5P054995 | 1.79225E-06 | 9.848130413 | down | noncoding | ENST00000528684 | FAM86C2P |
| ASHGA5P040082 | 0.000135236 | 9.801060086 | down | noncoding | ENST00000506942 | RP11-33B1.3 |
| ASHGA5P016092 | 0.000228497 | 9.711884965 | down | noncoding | uc010fjx.1 | LINC00116 |
| ASHGA5P041777 | 4.95661E-05 | 9.565011479 | down | noncoding | NR_037170 | LOC100507547 |
| ASHGA5P025371 | 0.000184079 | 9.516821862 | down | noncoding | NR_002921 | SNORA75 |
| ASHGA5P030010 | 8.98685E-05 | 9.482791179 | down | noncoding | ENST00000559134 | RP11-154J22.1 |
| ASHGA5P033159 | 0.000106738 | 9.458011745 | down | noncoding | ENST00000570002 | RP11-259G18.1 |
| ASHGA5P027965 | 0.011545946 | 9.443284923 | down | noncoding | ENST00000547866 | RP11-386G11.5 |
| ASHGA5P032911 | 5.66354E-06 | 9.34610341 | down | noncoding | ENST00000434303 | AC090286.2 |
| ASHGA5P030310 | 6.33987E-05 | 9.340395462 | down | noncoding | TCONS_00008646 | XLOC_004165 |
| ASHGA5P014027 | 0.002102745 | 9.318887277 | down | noncoding | ENST00000398804 | RP11-184I16.2 |
| ASHGA5P033688 | 0.001052861 | 9.248804105 | down | noncoding | ENST00000581677 | RP11-705O1.8 |
| ASHGA5P038730 | 0.000147281 | 9.209621722 | down | noncoding | uc001djh.1 | BC037304 |
| ASHGA5P036777 | 4.88239E-05 | 9.18992423 | down | noncoding | uc002xig.3 | LOC388796 |
| ASHGA5P041671 | 7.9074E-05 | 9.155792497 | down | noncoding | ENST00000566170 | RP1-223E5.4 |
| ASHGA5P047638 | 3.90651E-06 | 9.090784297 | down | noncoding | ENST00000454935 | LINC00263 |
| ASHGA5P051869 | 0.000963964 | 9.089383217 | down | noncoding | NR_024569 | LOC100130872 |
| ASHGA5P024093 | 1.07428E-06 | 9.048321137 | down | noncoding | hsa-mir-17 | MI0000071 |
| ASHGA5P032765 | 1.49373E-05 | 8.892022096 | down | noncoding | ENST00000575043 | CTD-3195I5.4 |
| ASHGA5P051168 | 7.82356E-06 | 8.879381779 | down | noncoding | ENST00000444998 | MCM3AP-AS1 |
| ASHGA5P018288 | 2.86454E-05 | 8.815873158 | down | noncoding | ENST00000451607 | GAS5 |
| ASHGA5P039588 | 0.000187899 | 8.801203814 | down | noncoding | ENST00000565254 | RP11-50D9.3 |
| ASHGA5P034832 | 0.000115668 | 8.784458965 | down | noncoding | ENST00000456949 | AC116614.1 |
| ASHGA5P016554 | 1.12971E-05 | 8.760138423 | down | noncoding | NR_049728 | PSMG1 |
| ASHGA5P043640 | 5.64498E-05 | 8.572992017 | down | noncoding | ENST00000441052 | AC017116.8 |
| ASHGA5P058062 | 6.54442E-05 | 8.510173235 | down | noncoding | TCONS_00026069 | XLOC_012467 |
| ASHGA5P025579 | 0.000237141 | 8.358472216 | down | noncoding | NR_003687 | SNORD19B |
| ASHGA5P018893 | 4.8567E-05 | 8.204284191 | down | noncoding | ENST00000484945 | FAM86DP |
| ASHGA5P019467 | 1.27749E-05 | 8.192239539 | down | noncoding | ENST00000486431 | RP11-666A20.1 |
| ASHGA5P021103 | 2.97241E-05 | 8.138852673 | down | noncoding | ENST00000525180 | FAM86C2P |
| ASHGA5P044133 | 8.41612E-06 | 8.106109515 | down | noncoding | uc011kxs.1 | DQ587039 |
| ASHGA5P019079 | 1.01371E-05 | 8.096564053 | down | noncoding | NR_024250 | FAM86JP |
| ASHGA5P053591 | 2.72967E-05 | 8.076971973 | down | noncoding | ENST00000524335 | RP13-582O9.5 |
| ASHGA5P040089 | 0.001543802 | 8.040191145 | down | noncoding | ENST00000508362 | RP11-679C8.2 |
| ASHGA5P046308 | 9.82509E-06 | 7.967759491 | down | noncoding | TCONS_00001546 | XLOC_000889 |
| ASHGA5P045716 | 0.000279866 | 7.961745743 | down | noncoding | ENST00000434871 | RP11-405L18.1 |
| ASHGA5P044370 | 0.000249156 | 7.923768329 | down | noncoding | ENST00000566000 | RP11-219B4.7 |
| ASHGA5P014184 | 0.000231399 | 7.844255715 | down | noncoding | NR_033255 | DTYMK |
| ASHGA5P054923 | 0.000107168 | 7.815873767 | down | noncoding | uc001nvr.3 | SNHG1 |
| ASHGA5P041415 | 0.001214609 | 7.801066655 | down | noncoding | NR_026704 | VTRNA1-2 |
| ASHGA5P045498 | 9.18976E-05 | 7.772743456 | down | noncoding | uc004bwk.3 | BC022468 |
| ASHGA5P026856 | 0.000962821 | 7.698108472 | down | noncoding | TCONS_00011856 | XLOC_005347 |
| ASHGA5P047359 | 0.000570538 | 7.583415126 | down | noncoding | ENST00000429214 | RP11-162G10.5 |
| ASHGA5P045717 | 7.04836E-05 | 7.561435943 | down | noncoding | uc004aaa.3 | BC014180 |
| ASHGA5P015833 | 0.000242757 | 7.515197749 | down | noncoding | NR_024534 | ALG3 |
| ASHGA5P029325 | 5.58413E-05 | 7.484695028 | down | noncoding | ENST00000500370 | SNHG10 |
| ASHGA5P048762 | 0.007029021 | 7.48292129 | down | noncoding | NR_040055 | IQCH-AS1 |
| ASHGA5P006110 | 2.21654E-05 | 7.477413417 | down | noncoding | uc003ghn.3 | FAM86EP |
| ASHGA5P043361 | 9.66983E-05 | 7.47259103 | down | noncoding | NR_033999 | LOC100129148 |
| ASHGA5P017314 | 0.000140193 | 7.463033869 | down | noncoding | NR_037669 | GGCT |
| ASHGA5P044134 | 0.000299419 | 7.45569495 | down | noncoding | uc003wvp.1 | AK308439 |
| ASHGA5P032940 | 4.81394E-06 | 7.454526237 | down | noncoding | ENST00000578585 | RP11-283C24.1 |
| ASHGA5P025349 | 1.55276E-06 | 7.444296064 | down | noncoding | NR_002739 | SNORD56 |
| ASHGA5P025298 | 0.000738917 | 7.410301969 | down | noncoding | NR_002325 | SNORA6 |
| ASHGA5P017358 | 2.25959E-06 | 7.408091921 | down | noncoding | ENST00000440568 | AC034193.5 |
| ASHGA5P044052 | 2.29525E-05 | 7.40020276 | down | noncoding | uc003woi.3 | AK021933 |
| ASHGA5P045563 | 0.000222717 | 7.391093545 | down | noncoding | ENST00000434532 | RP11-229P13.21 |
| ASHGA5P051944 | 0.000313429 | 7.377134129 | down | noncoding | NR_024031 | DANCR |
| ASHGA5P025429 | 0.000537609 | 7.368544635 | down | noncoding | NR_003006 | SCARNA6 |
| ASHGA5P057645 | 8.08456E-05 | 7.34667659 | down | noncoding | TCONS_00020346 | XLOC_009677 |
| ASHGA5P048185 | 0.000380442 | 7.267631648 | down | noncoding | ENST00000545308 | SNHG1 |
| ASHGA5P047581 | 6.59014E-05 | 7.249432018 | down | noncoding | uc001kgu.3 | KRMP1 |
| ASHGA5P047776 | 0.0016121 | 7.145937356 | down | noncoding | TCONS_00013639 | XLOC_006301 |
| ASHGA5P053705 | 0.002322953 | 7.136699695 | down | noncoding | ENST00000315762 | RP11-12A20.7 |
| ASHGA5P022774 | 0.004235337 | 7.101723051 | down | noncoding | ENST00000562555 | RP11-259G18.1 |
| ASHGA5P012461 | 4.15127E-05 | 7.091596738 | down | noncoding | NR_034181 | SUV39H2 |
| ASHGA5P038421 | 0.000221082 | 7.045636769 | down | noncoding | uc003ene.2 | FAM86HP |
| ASHGA5P043000 | 0.000297362 | 6.997953282 | down | noncoding | uc003tep.1 | DPY19L2P1 |
| ASHGA5P031297 | 3.71187E-05 | 6.995325068 | down | noncoding | ENST00000436656 | GAS5 |
| ASHGA5P031297 | 3.71187E-05 | 6.995325068 | down | noncoding | ENST00000436656 | GAS5 |
| ASHGA5P048186 | 0.000217109 | 6.957832748 | down | noncoding | uc001nvs.3 | SNHG1 |
| ASHGA5P041905 | 4.83486E-05 | 6.956225333 | down | noncoding | NR_027005 | C6orf147 |
| ASHGA5P047922 | 4.05022E-06 | 6.949506183 | down | noncoding | ENST00000525154 | RP11-540A21.2 |
| ASHGA5P036838 | 4.33983E-06 | 6.907479299 | down | noncoding | TCONS_00025860 | XLOC_012138 |
| ASHGA5P051945 | 2.80444E-05 | 6.903353362 | down | noncoding | ENST00000425653 | DANCR |
| ASHGA5P025333 | 5.54712E-06 | 6.901432276 | down | noncoding | NR_002584 | SNORA69 |
| ASHGA5P058042 | 0.000242263 | 6.854230775 | down | noncoding | TCONS_00025631 | XLOC_012467 |
| ASHGA5P029329 | 4.92946E-05 | 6.839475374 | down | noncoding | ENST00000569214 | RP11-872J21.3 |
| ASHGA5P048174 | 0.000441403 | 6.809852421 | down | noncoding | ENST00000525500 | RP11-838H22.2 |
| ASHGA5P044460 | 0.000535386 | 6.796896401 | down | noncoding | ENST00000577199 | RP11-1C8.6 |
| ASHGA5P037342 | 2.83129E-05 | 6.777875327 | down | noncoding | uc002zcj.2 | AX747730 |
| ASHGA5P056691 | 5.25176E-05 | 6.766924693 | down | noncoding | TCONS_00007460 | XLOC_003427 |
| ASHGA5P032686 | 5.5853E-05 | 6.75011306 | down | noncoding | ENST00000562672 | CTD-2526A2.2 |
| ASHGA5P039926 | 0.020885245 | 6.737618615 | down | noncoding | ENST00000503051 | USP46-AS1 |
| ASHGA5P025093 | 0.000267142 | 6.7124596 | down | noncoding | hsa-mir-664b | MI0019134 |
| ASHGA5P033778 | 1.41721E-05 | 6.668313815 | down | noncoding | ENST00000577906 | RP11-13N13.2 |
| ASHGA5P014621 | 0.000174263 | 6.665256211 | down | noncoding | NR_024533 | ALG3 |
| ASHGA5P039115 | 0.000576861 | 6.65583099 | down | noncoding | uc003faz.1 | AK094480 |
| ASHGA5P053882 | 6.39785E-05 | 6.653719586 | down | noncoding | uc004cin.3 | SNHG7 |
| ASHGA5P019542 | 0.000441604 | 6.646989001 | down | noncoding | NR_046295 | MSTO1 |
| ASHGA5P025420 | 0.000407838 | 6.642807312 | down | noncoding | NR_002997 | SCARNA1 |
| ASHGA5P027137 | 0.000472537 | 6.63115832 | down | noncoding | ENST00000545819 | RP11-283G6.3 |
| ASHGA5P025697 | 7.75438E-05 | 6.628928996 | down | noncoding | NR_027083 | AFMID |
| ASHGA5P025697 | 7.75438E-05 | 6.628928996 | down | noncoding | NR_027083 | AFMID |
| ASHGA5P042860 | 1.1656E-06 | 6.577718798 | down | noncoding | TCONS_00019861 | XLOC_009079 |
| ASHGA5P048735 | 0.001177909 | 6.567697895 | down | noncoding | uc001bql.3 | SNHG12 |
| ASHGA5P045455 | 0.000964756 | 6.50601899 | down | noncoding | TCONS_00000890 | XLOC_000141 |
| ASHGA5P022540 | 0.000618786 | 6.500421342 | down | noncoding | ENST00000561384 | CTD-2008A1.2 |
| ASHGA5P041961 | 0.001944035 | 6.490182891 | down | noncoding | TCONS_00020260 | XLOC_010236 |
| ASHGA5P020573 | 0.000576047 | 6.47516789 | down | noncoding | ENST00000516404 | SNORA74 |
| ASHGA5P031715 | 0.0001511 | 6.463398454 | down | noncoding | TCONS_00005763 | XLOC_003092 |
| ASHGA5P039217 | 0.000320035 | 6.441295894 | down | noncoding | ENST00000434957 | RP11-573D15.3 |
| ASHGA5P029803 | 0.000567228 | 6.427426616 | down | noncoding | ENST00000560931 | RP11-365N19.2 |
| ASHGA5P042969 | 2.61812E-05 | 6.422925518 | down | noncoding | uc003tad.4 | LOC646762 |
| ASHGA5P022174 | 0.000248457 | 6.392245719 | down | noncoding | ENST00000551152 | RP11-396F22.1 |
| ASHGA5P025385 | 0.000498329 | 6.368569121 | down | noncoding | NR_002962 | SNORA23 |
| ASHGA5P045406 | 2.65892E-05 | 6.360819876 | down | noncoding | ENST00000440009 | RP11-276E15.4 |
| ASHGA5P033068 | 0.000839287 | 6.343161466 | down | noncoding | ENST00000563897 | CTB-58E17.1 |
| ASHGA5P044836 | 5.11639E-05 | 6.343015349 | down | noncoding | TCONS_00002142 | XLOC_000363 |
| ASHGA5P025732 | 0.000150197 | 6.337800877 | down | noncoding | NR_028581 | G6PC3 |
| ASHGA5P057289 | 0.000598931 | 6.330811471 | down | noncoding | TCONS_00015403 | XLOC_006993 |
| ASHGA5P019295 | 5.46127E-06 | 6.322497085 | down | noncoding | ENST00000477247 | FAM86DP |
| ASHGA5P017159 | 0.000108462 | 6.30929555 | down | noncoding | ENST00000438380 | RP11-175D17.3 |
| ASHGA5P018565 | 5.1741E-05 | 6.295175651 | down | noncoding | uc001vvv.1 | AK056135 |
| ASHGA5P019861 | 3.39717E-06 | 6.258814856 | down | noncoding | ENST00000504277 | RP11-159F24.5 |
| ASHGA5P047781 | 4.97581E-06 | 6.247924558 | down | noncoding | TCONS_00013643 | XLOC_006301 |
| ASHGA5P025405 | 2.2085E-05 | 6.239534784 | down | noncoding | NR_002982 | SNORA54 |
| ASHGA5P025403 | 0.000946448 | 6.205302035 | down | noncoding | NR_002980 | SNORA50 |
| ASHGA5P036587 | 0.001081071 | 6.204073164 | down | noncoding | ENST00000439601 | AC131097.3 |
| ASHGA5P023454 | 0.00046223 | 6.184838398 | down | noncoding | ENST00000581248 | RP11-599B13.7 |
| ASHGA5P032055 | 0.000117527 | 6.184567323 | down | noncoding | ENST00000570809 | RP11-676J12.4 |
| ASHGA5P025392 | 0.000426179 | 6.180504328 | down | noncoding | NR_002969 | SNORA36A |
| ASHGA5P030499 | 0.000219961 | 6.163888935 | down | noncoding | uc001zsy.3 | BC062349 |
| ASHGA5P037731 | 1.98436E-05 | 6.128328785 | down | noncoding | ENST00000566851 | RRP7B |
| ASHGA5P025474 | 0.000770381 | 6.117459026 | down | noncoding | NR_003069 | SNORD88C |
| ASHGA5P025369 | 0.003261328 | 6.116915585 | down | noncoding | NR_002919 | SNORA5A |
| ASHGA5P038918 | 0.023194414 | 6.116170817 | down | noncoding | uc003dtq.3 | AF090939 |
| ASHGA5P015604 | 0.001155747 | 6.102994823 | down | noncoding | ENST00000421848 | RP11-374M1.4 |
| ASHGA5P031682 | 0.006111455 | 6.060782065 | down | noncoding | ENST00000565152 | RP11-388M20.1 |
| ASHGA5P039312 | 2.50812E-05 | 6.051096113 | down | noncoding | ENST00000510506 | FAM86EP |
| ASHGA5P024108 | 0.000599672 | 6.045174144 | down | noncoding | hsa-mir-18a | MI0000072 |
| ASHGA5P025410 | 4.49709E-05 | 6.03537048 | down | noncoding | NR_002987 | SNORA61 |
| ASHGA5P053406 | 5.84289E-05 | 6.022711141 | down | noncoding | ENST00000442067 | GAS5 |
| ASHGA5P016667 | 0.002593141 | 6.009750649 | down | noncoding | ENST00000432735 | MCM3AP-AS1 |
| ASHGA5P038690 | 0.010411374 | 5.991689516 | down | noncoding | ENST00000326237 | AC034193.5 |
| ASHGA5P013052 | 0.000196371 | 5.975782475 | down | noncoding | uc002zkv.3 | AK056135 |
| ASHGA5P031457 | 9.51541E-05 | 5.9700727 | down | noncoding | ENST00000452867 | RP11-21M7.1 |
| ASHGA5P007972 | 5.31265E-06 | 5.944439837 | down | noncoding | ENST00000338711 | FAM86FP |
| ASHGA5P025473 | 0.000271592 | 5.937744984 | down | noncoding | NR_003068 | SNORD88B |
| ASHGA5P032878 | 9.47024E-05 | 5.927873249 | down | noncoding | ENST00000480811 | C17orf76-AS1 |
| ASHGA5P041416 | 0.000703631 | 5.916121849 | down | noncoding | NR_026705 | VTRNA1-3 |
| ASHGA5P047637 | 1.3993E-05 | 5.898058506 | down | noncoding | NR_026762 | LINC00263 |
| ASHGA5P038013 | 8.0102E-06 | 5.895405301 | down | noncoding | NR_024355 | LINC00634 |
| ASHGA5P037800 | 0.000101487 | 5.872204998 | down | noncoding | ENST00000565162 | AC004463.6 |
| ASHGA5P044519 | 0.000185821 | 5.872144079 | down | noncoding | ENST00000523030 | RP11-6D1.3 |
| ASHGA5P040106 | 1.53278E-05 | 5.846336759 | down | noncoding | ENST00000413650 | AL583842.3 |
| ASHGA5P018118 | 0.00062084 | 5.824263391 | down | noncoding | ENST00000449648 | RP11-5N23.2 |
| ASHGA5P030309 | 1.77853E-05 | 5.811429167 | down | noncoding | ENST00000560221 | RP11-35O15.1 |
| ASHGA5P024135 | 0.000132769 | 5.770354421 | down | noncoding | hsa-mir-19a | MI0000073 |
| ASHGA5P019333 | 0.000126299 | 5.766045682 | down | noncoding | ENST00000478666 | FAM86DP |
| ASHGA5P022539 | 0.001990579 | 5.765854109 | down | noncoding | ENST00000558556 | CTD-2008A1.2 |
| ASHGA5P031284 | 2.47431E-05 | 5.765771513 | down | noncoding | ENST00000444470 | GAS5 |
| ASHGA5P025359 | 5.27637E-05 | 5.758692948 | down | noncoding | NR_002785 | GNAS-AS1 |
| ASHGA5P029450 | 2.77799E-05 | 5.752128878 | down | noncoding | TCONS_00016163 | XLOC_007573 |
| ASHGA5P053509 | 0.001048632 | 5.746558846 | down | noncoding | ENST00000419458 | RP11-21M7.1 |
| ASHGA5P033943 | 7.09753E-05 | 5.716986697 | down | noncoding | ENST00000501448 | AC005329.7 |
| ASHGA5P031556 | 0.000161927 | 5.668076775 | down | noncoding | ENST00000566734 | RP11-1021N1.2 |
| ASHGA5P048256 | 1.26722E-05 | 5.660484983 | down | noncoding | ENST00000529253 | FAM86C2P |
| ASHGA5P054916 | 6.44164E-05 | 5.650307694 | down | noncoding | ENST00000535076 | SNHG1 |
| ASHGA5P043628 | 9.06923E-05 | 5.646886497 | down | noncoding | NR_040085 | LOC100506776 |
| ASHGA5P015915 | 1.10333E-05 | 5.643901854 | down | noncoding | ENST00000424756 | LINC00539 |
| ASHGA5P032143 | 0.002713436 | 5.640362945 | down | noncoding | ENST00000577807 | RP11-599B13.3 |
| ASHGA5P051732 | 7.40071E-05 | 5.616707861 | down | noncoding | NR_027954 | TIPARP-AS1 |
| ASHGA5P037630 | 9.47921E-06 | 5.585337269 | down | noncoding | TCONS_00025630 | XLOC_012467 |
| ASHGA5P055829 | 0.000212358 | 5.576527617 | down | noncoding | uc001vmm.3 | AK094990 |
| ASHGA5P033086 | 0.000312631 | 5.5493227 | down | noncoding | uc001hta.3 | BC015435 |
| ASHGA5P025440 | 9.99493E-05 | 5.535170025 | down | noncoding | NR_003017 | SNORA71C |
| ASHGA5P016921 | 3.48108E-05 | 5.535019501 | down | noncoding | ENST00000435718 | RP4-564F22.2 |
| ASHGA5P021049 | 7.32923E-05 | 5.53414956 | down | noncoding | ENST00000523992 | RP11-556O5.3 |
| ASHGA5P056135 | 6.21994E-06 | 5.5317393 | down | noncoding | NR_001459 | SNHG10 |
| ASHGA5P051589 | 1.38472E-05 | 5.52119568 | down | noncoding | ENST00000489609 | FAM86DP |
| ASHGA5P046293 | 0.007847856 | 5.51158616 | down | noncoding | ENST00000563601 | RP11-390B4.5 |
| ASHGA5P050985 | 0.002303151 | 5.481755569 | down | noncoding | uc002xir.1 | SNHG11 |
| ASHGA5P027536 | 0.025374785 | 5.479058852 | down | noncoding | ENST00000550029 | RP11-341G23.4 |
| ASHGA5P036779 | 0.001048684 | 5.472884209 | down | noncoding | ENST00000439912 | SNORA71A |
| ASHGA5P039114 | 0.002982519 | 5.469629709 | down | noncoding | ENST00000488745 | SNHG12 |
| ASHGA5P034044 | 0.003035345 | 5.428249375 | down | noncoding | ENST00000398216 | RP11-54O7.2 |
| ASHGA5P048255 | 0.000161001 | 5.426935768 | down | noncoding | ENST00000531806 | FAM86C2P |
| ASHGA5P022588 | 0.000941813 | 5.425166945 | down | noncoding | NR_034039 | SORD |
| ASHGA5P035690 | 0.000172184 | 5.416589653 | down | noncoding | ENST00000455579 | AC010729.1 |
| ASHGA5P039289 | 1.83821E-05 | 5.403968506 | down | noncoding | NR_036511 | LOC100129917 |
| ASHGA5P047662 | 0.000159599 | 5.398824925 | down | noncoding | NR_038938 | LOC100505761 |
| ASHGA5P038926 | 0.001528401 | 5.396074001 | down | noncoding | uc003dwh.1 | AX747913 |
| ASHGA5P031959 | 0.000109065 | 5.391456242 | down | noncoding | ENST00000565382 | RP11-505K9.1 |
| ASHGA5P021764 | 0.004418341 | 5.389939826 | down | noncoding | ENST00000540369 | RP11-728G15.1 |
| ASHGA5P022077 | 3.85511E-06 | 5.379016198 | down | noncoding | NR_037592 | AK2 |
| ASHGA5P051924 | 0.000162667 | 5.374179219 | down | noncoding | ENST00000507033 | RP11-472B18.1 |
| ASHGA5P025362 | 0.001060203 | 5.365129521 | down | noncoding | NR_002911 | SNORA71A |
| ASHGA5P017289 | 0.003488065 | 5.362342615 | down | noncoding | ENST00000439760 | RP11-374M1.2 |
| ASHGA5P025591 | 0.000306452 | 5.361172293 | down | noncoding | NR_003705 | SNORA36C |
| ASHGA5P019993 | 0.001043619 | 5.35866923 | down | noncoding | ENST00000506448 | FAM86HP |
| ASHGA5P024249 | 0.000454213 | 5.354345866 | down | noncoding | hsa-mir-3143 | MI0014167 |
| ASHGA5P051848 | 0.000615474 | 5.352616656 | down | noncoding | ENST00000452051 | TM4SF19-AS1 |
| ASHGA5P019460 | 0.000787218 | 5.348664208 | down | noncoding | NR_024251 | FAM86JP |
| ASHGA5P055188 | 0.003972872 | 5.341189733 | down | noncoding | ENST00000577297 | RP11-305N23.1 |
| ASHGA5P058041 | 0.007169746 | 5.339149083 | down | noncoding | TCONS_00025619 | XLOC_012456 |
| ASHGA5P053495 | 2.39868E-06 | 5.305468056 | down | noncoding | ENST00000501194 | RP11-68L18.1 |
| ASHGA5P025620 | 0.003620988 | 5.297684276 | down | noncoding | NR_004403 | SNORD97 |
| ASHGA5P017607 | 0.00089977 | 5.285600917 | down | noncoding | NR_033338 | C17orf70 |
| ASHGA5P039618 | 0.000276356 | 5.283016198 | down | noncoding | ENST00000514727 | PPP1R14BP3 |
| ASHGA5P000103 | 8.55395E-06 | 5.263695258 | down | noncoding | BC064139 |  |
| ASHGA5P024356 | 0.001362776 | 5.254994788 | down | noncoding | hsa-mir-3651 | MI0016051 |
| ASHGA5P014947 | 0.038971165 | 5.236713341 | down | noncoding | ENST00000415062 | RP11-426A6.5 |
| ASHGA5P036258 | 0.000163499 | 5.228157608 | down | noncoding | TCONS_00026882 | XLOC_012945 |
| ASHGA5P026916 | 0.000282286 | 5.217629368 | down | noncoding | NR_024233 | LINC00167 |
| ASHGA5P025617 | 0.00680571 | 5.204598475 | down | noncoding | NR_004397 | SNORD1C |
| ASHGA5P053588 | 0.000314366 | 5.195475764 | down | noncoding | ENST00000523031 | RP13-582O9.5 |
| ASHGA5P031935 | 0.000162965 | 5.187795702 | down | noncoding | ENST00000563230 | RP11-556H2.3 |
| ASHGA5P052171 | 1.58223E-05 | 5.187526016 | down | noncoding | uc021xvv.1 | AK023178 |
| ASHGA5P028433 | 7.91145E-05 | 5.15881949 | down | noncoding | ENST00000449656 | C1QTNF9-AS1 |
| ASHGA5P025446 | 7.0938E-05 | 5.138176089 | down | noncoding | NR_003026 | SNORA1 |
| ASHGA5P044042 | 0.003048139 | 5.134887492 | down | noncoding | NR_038835 | LOC645249 |
| ASHGA5P017648 | 2.17383E-07 | 5.121777331 | down | noncoding | ENST00000444126 | HCG18 |
| ASHGA5P053474 | 0.004830876 | 5.112053495 | down | noncoding | ENST00000518880 | RP11-363E6.3 |
| ASHGA5P039787 | 2.40543E-05 | 5.09152853 | down | noncoding | ENST00000514763 | RP11-323F5.2 |
| ASHGA5P025432 | 0.002468758 | 5.066596717 | down | noncoding | NR_003009 | SCARNA8 |
| ASHGA5P025346 | 0.000203596 | 5.065374724 | down | noncoding | NR_002736 | SNORD60 |
| ASHGA5P051170 | 0.001727179 | 5.06414191 | down | noncoding | ENST00000447037 | AP001469.9 |
| ASHGA5P022161 | 0.001988505 | 5.043210348 | down | noncoding | ENST00000550853 | LINC00094 |
| ASHGA5P045580 | 3.73238E-05 | 5.033770003 | down | noncoding | TCONS_00001800 | XLOC_001189 |
| ASHGA5P019192 | 0.000172232 | 5.031920631 | down | noncoding | NR_028444 | PDIA5 |
| ASHGA5P025602 | 0.000158066 | 5.022348707 | down | noncoding | NR_003942 | SNORD76 |
| ASHGA5P031057 | 0.000308748 | 5.022069869 | down | noncoding | ENST00000567731 | RP11-22P6.2 |
| ASHGA5P048987 | 0.006836394 | 5.015433374 | down | noncoding | uc010brs.1 | BC114455 |
| ASHGA5P031059 | 0.000607053 | 5.003987781 | down | noncoding | NR_046289 | LOC100289092 |
