# Supplementary material for: LAST, a c-Myc-inducible long noncoding RNA, cooperates with CNBP to promote CCND1 mRNA stability in human cells
Source: eLife. 2017 Dec 4;6:e30433. doi: 10.7554/eLife.30433 (PMC5739540; doi:10.7554/eLife.30433)
Supplement: Supplementary file 2. — Information of SOX9, PDF, NFE2L1 and CCND1 is colored. [file elife-30433-supp2.doc]

**Supplementary file 2. Overlap of CNBP RIP sequencing dataset and *LAST* knockdown mRNA sequencing dataset.**

**^a^ Log2(fold change), if the comparison is test vs control, log2 of the fold change will be calculated by log2(Test FPKM+1)-log2(Control FPKM+1).**

**^b^ Fold Change, 2^(log2(fold change)).**

|  | **Down**  **sh-LAST-1 vs sh-ctrl**  **(gene level)** | | **Up**  **RIP vs input**  **(gene level)** | |
| --- | --- | --- | --- | --- |
| **Gene Name** | **log2(fold change)^a^** | **Fold Change^b^** | **log2(fold change)^a^** | **Fold Change^b^** |
| **EVPL** | **-0.593675735** | **0.662652432** | **5.534498928** | **46.35004801** |
| **SOX9** | **-0.585305081** | **0.66650838** | **4.463072807** | **22.05559548** |
| **CBX6** | **-2.506179461** | **0.176021131** | **4.237792686** | **18.86699412** |
| **PDF** | **-2.557267547** | **0.16989702** | **4.075780589** | **16.862898** |
| **TBC1D16** | **-0.610537604** | **0.654952596** | **3.991158635** | **15.90224596** |
| **HIST2H2AA3** | **-1.101791246** | **0.46593763** | **3.894625918** | **14.8730221** |
| **JUND** | **-3.441895571** | **0.09202084** | **3.882612044** | **14.74968301** |
| **IRS1** | **-0.818337147** | **0.567095201** | **3.739567894** | **13.35740539** |
| **HS6ST1** | **-3.618560865** | **0.08141504** | **3.68815521** | **12.88977531** |
| **ACBD3** | **-1.008306563** | **0.497129437** | **3.646980849** | **12.52710245** |
| **SOCS7** | **-1.447971243** | **0.366536495** | **3.553166969** | **11.73842521** |
| **THRAP3** | **-0.651202198** | **0.636749489** | **3.503160138** | **11.33851767** |
| **NFE2L1** | **-0.980428021** | **0.506829351** | **3.41525434** | **10.66827001** |
| **SOX4** | **-3.157506425** | **0.112071673** | **3.348455683** | **10.18557614** |
| **ANKRD33B** | **-2.731169618** | **0.150603832** | **3.177210337** | **9.045563235** |
| **SETX** | **-0.632583958** | **0.645020108** | **3.06816839** | **8.387078677** |
| **C6orf106** | **-2.124386374** | **0.22934854** | **3.034049847** | **8.191058209** |
| **MSN** | **-1.222531951** | **0.428529981** | **3.01164839** | **8.064853853** |
| **SUDS3** | **-1.164334593** | **0.446169997** | **2.940968252** | **7.679265097** |
| **EPHA2** | **-0.628682478** | **0.646766797** | **2.86719579** | **7.296455467** |
| **TNFRSF10B** | **-0.965605525** | **0.512063444** | **2.841250759** | **7.166410868** |
| **VCL** | **-0.869150478** | **0.54746913** | **2.841182257** | **7.1660706** |
| **AMOTL2** | **-1.561712444** | **0.338748757** | **2.808726105** | **7.006656183** |
| **GPX1** | **-1.467713164** | **0.361554951** | **2.786039655** | **6.897337945** |
| **SSH1** | **-1.590460079** | **0.33206554** | **2.77453301** | **6.842544966** |
| **ZFR** | **-0.746410302** | **0.596084886** | **2.772511475** | **6.832963761** |
| **SRSF1** | **-0.676256877** | **0.625786796** | **2.749356221** | **6.724170101** |
| **NR2F1** | **-0.687271943** | **0.621027069** | **2.748101917** | **6.718326535** |
| **FANCE** | **-0.80433419** | **0.572626289** | **2.725060024** | **6.61187763** |
| **METTL7B** | **-0.849750035** | **0.554880868** | **2.707462012** | **6.531715767** |
| **HIP1** | **-1.423020943** | **0.372930593** | **2.700556948** | **6.500528195** |
| **RHOBTB2** | **-0.802946934** | **0.573177175** | **2.699447416** | **6.495530765** |
| **KCNJ11** | **-0.626475722** | **0.647756853** | **2.689040476** | **6.448843577** |
| **ADNP** | **-1.74017665** | **0.299333022** | **2.67626932** | **6.392008468** |
| **ATP13A3** | **-1.037536359** | **0.487158668** | **2.634068501** | **6.207741577** |
| **FKBP9** | **-0.993399896** | **0.502292662** | **2.609902164** | **6.104622837** |
| **ZNF574** | **-0.910728696** | **0.531916356** | **2.566981831** | **5.925684566** |
| **FAM203B** | **-0.860723957** | **0.550676154** | **2.530962293** | **5.77957053** |
| **AC007192.4** | **-1.981137618** | **0.253290063** | **2.527149413** | **5.764315948** |
| **ZNF618** | **-1.12344922** | **0.458995139** | **2.485965316** | **5.602090601** |
| **CDR2** | **-1.584866117** | **0.333355603** | **2.471823162** | **5.547443858** |
| **SFPQ** | **-0.59736088** | **0.660961947** | **2.380951135** | **5.20880033** |
| **MRFAP1** | **-1.568658321** | **0.337121765** | **2.368064059** | **5.162479182** |
| **CSNK2A3** | **-4.185901978** | **0.054943706** | **2.357646305** | **5.125335** |
| **KHDRBS1** | **-0.62854672** | **0.646827661** | **2.347966938** | **5.091063069** |
| **MSH6** | **-1.22715894** | **0.427157808** | **2.323235728** | **5.004533966** |
| **ANKRD40** | **-1.635798957** | **0.321792151** | **2.295463457** | **4.909116678** |
| **TFRC** | **-0.908700697** | **0.532664597** | **2.278512898** | **4.851775844** |
| **DENND6A** | **-0.690543485** | **0.619620386** | **2.271028448** | **4.826670854** |
| **KLHL8** | **-1.267721257** | **0.415315247** | **2.248556182** | **4.752070312** |
| **AMOTL1** | **-1.279589867** | **0.411912591** | **2.244698341** | **4.739380003** |
| **HIST1H2BL** | **-0.662663764** | **0.631710841** | **2.237519438** | **4.715855255** |
| **SH3BP4** | **-0.916845294** | **0.529665962** | **2.218812453** | **4.655100946** |
| **KIDINS220** | **-0.747819812** | **0.595502797** | **2.209417666** | **4.624885557** |
| **RAD21** | **-1.005061512** | **0.498248887** | **2.207719704** | **4.619445556** |
| **ARHGAP11A** | **-1.240711143** | **0.423164016** | **2.206501631** | **4.615546984** |
| **VSIG10** | **-0.940317804** | **0.521118073** | **2.202192607** | **4.601781895** |
| **CCAR2** | **-0.699340451** | **0.615853689** | **2.189110257** | **4.56024159** |
| **NPTXR** | **-1.375180433** | **0.385504489** | **2.177857112** | **4.52480968** |
| **GPD2** | **-0.992578323** | **0.502578785** | **2.177293935** | **4.5230437** |
| **HIF1AN** | **-1.513649167** | **0.350224237** | **2.141133324** | **4.411084275** |
| **EGR1** | **-0.975850994** | **0.508439847** | **2.133042519** | **4.386415629** |
| **CHD6** | **-0.663852503** | **0.631190544** | **2.123160392** | **4.356472366** |
| **CCND1** | **-0.775416802** | **0.584219816** | **2.118215588** | **4.341566218** |
| **AFAP1** | **-0.808673949** | **0.570906365** | **2.103655335** | **4.297969769** |
| **NUDT16** | **-1.447709958** | **0.366602884** | **2.099384135** | **4.285264145** |
| **CRLF3** | **-1.265263203** | **0.416023462** | **2.0970597** | **4.278365391** |
| **C19orf43** | **-1.310730047** | **0.403116839** | **2.051726988** | **4.146019748** |
| **SPECC1L** | **-0.73947738** | **0.598956287** | **2.050988384** | **4.143897689** |
| **CHST7** | **-1.269245045** | **0.414876819** | **2.039229712** | **4.110260158** |
| **ONECUT3** | **-0.947486393** | **0.518535119** | **2.031396289** | **4.088003096** |
| **ITPRIP** | **-0.646647236** | **0.638763049** | **2.023560141** | **4.065858876** |
| **FNBP1** | **-0.805116405** | **0.572315901** | **2.015885206** | **4.044286509** |
| **ZBTB3** | **-0.818691398** | **0.566955969** | **2.007702297** | **4.021412408** |
| **TRIM35** | **-1.503367149** | **0.352729184** | **2.00143097** | **4.00396946** |
| RAD54L2 | -1.38150504 | 0.383818182 | 1.98237736 | 3.951436873 |
| ZNF469 | -1.155134439 | 0.449024342 | 1.974973784 | 3.931210957 |
| C18orf25 | -1.452810108 | 0.365309175 | 1.919379817 | 3.782604179 |
| DDX21 | -1.167616435 | 0.445156203 | 1.916359124 | 3.774692505 |
| CAND1 | -0.788647087 | 0.578886699 | 1.915866292 | 3.773403271 |
| TMEM65 | -1.623183969 | 0.324618253 | 1.890192348 | 3.706846432 |
| PHIP | -0.930548876 | 0.524658696 | 1.853754614 | 3.614396088 |
| CUEDC1 | -0.822913715 | 0.565299092 | 1.847638941 | 3.599106869 |
| MALT1 | -0.971755879 | 0.509885113 | 1.843216977 | 3.588092237 |
| SYNM | -0.605249425 | 0.657357721 | 1.835890179 | 3.569916126 |
| SNTB2 | -1.901622687 | 0.267642163 | 1.827768486 | 3.549875643 |
| ZNF217 | -1.156894676 | 0.44847682 | 1.827173727 | 3.548412488 |
| ZBED1 | -0.738304506 | 0.599443421 | 1.804238871 | 3.492448572 |
| KRAS | -2.080678816 | 0.236403153 | 1.801219353 | 3.485146625 |
| TRAF3IP1 | -0.665440635 | 0.630496106 | 1.789968607 | 3.458073676 |
| OSBPL11 | -0.924755657 | 0.526769727 | 1.776448803 | 3.425818699 |
| SP6 | -0.650350376 | 0.637125561 | 1.759379635 | 3.385525147 |
| AARS | -0.630597768 | 0.645908733 | 1.757767273 | 3.381743583 |
| TAOK1 | -1.063948063 | 0.478321298 | 1.748707225 | 3.360572958 |
| WDR82 | -1.879110583 | 0.271851259 | 1.731298032 | 3.320264171 |
| RAB11FIP2 | -1.396032038 | 0.379972776 | 1.717663318 | 3.289032624 |
| APEX2 | -1.483340414 | 0.357659728 | 1.690545344 | 3.227786922 |
| MOCS3 | -2.490719526 | 0.177917518 | 1.665872528 | 3.173055 |
| BMP2K | -0.605836933 | 0.65709008 | 1.645703438 | 3.129003859 |
| RBM15B | -3.16229577 | 0.111700243 | 1.63386578 | 3.103434678 |
| CNST | -0.67982978 | 0.624238923 | 1.617509391 | 3.068448546 |
| PPP4R3B | -0.769995916 | 0.586419135 | 1.61406981 | 3.06114166 |
| DCBLD2 | -1.283994015 | 0.410657054 | 1.608835466 | 3.05005544 |
| ZBED3 | -0.799953179 | 0.574367818 | 1.598270021 | 3.02780023 |
| FAM155B | -1.646349751 | 0.319447388 | 1.581743744 | 2.993314245 |
| BMP8B | -0.730575628 | 0.602663406 | 1.540358535 | 2.9086678 |
| EID1 | -1.505242337 | 0.352271011 | 1.539157853 | 2.90624807 |
| HECTD3 | -0.787881975 | 0.579193784 | 1.536016529 | 2.89992689 |
| CCDC43 | -0.920206183 | 0.528433494 | 1.530313796 | 2.888486589 |
| HMG20A | -1.13853735 | 0.454219847 | 1.505008685 | 2.838263799 |
| SH3BP5L | -0.628046065 | 0.647052166 | 1.476063522 | 2.781886427 |
| SYT1 | -0.594159965 | 0.662430055 | 1.453973094 | 2.739614865 |
| ATXN7L3B | -3.466079823 | 0.090491129 | 1.443152409 | 2.719143724 |
| TOP2A | -0.648644449 | 0.637879381 | 1.441577522 | 2.716177049 |
| GNS | -1.164352707 | 0.446164395 | 1.435003551 | 2.70382832 |
| TMEM200B | -0.60614082 | 0.656951686 | 1.422184422 | 2.679909761 |
| ZFX | -0.607822908 | 0.65618617 | 1.417388785 | 2.671016309 |
| PSPC1 | -0.741651833 | 0.59805421 | 1.390981846 | 2.622571028 |
| NUDT4 | -2.179976394 | 0.22067936 | 1.373876901 | 2.591660785 |
| CACUL1 | -1.649854069 | 0.318672389 | 1.366243261 | 2.577983913 |
| TGFBR1 | -1.446388974 | 0.366938713 | 1.361835411 | 2.570119448 |
| GAREM2 | -0.883518326 | 0.542043926 | 1.359715566 | 2.566345779 |
| RRM2B | -0.59205334 | 0.663398042 | 1.359474283 | 2.565916606 |
| TNPO3 | -0.748051793 | 0.595407049 | 1.341410933 | 2.533990179 |
| CDCA2 | -0.794274048 | 0.576633258 | 1.34140124 | 2.533973155 |
| STRN3 | -0.729844381 | 0.602968951 | 1.340137911 | 2.531755194 |
| ZBED1 | -0.738304506 | 0.599443421 | 1.33339601 | 2.519951575 |
| TMEM158 | -1.805734877 | 0.286035303 | 1.319113828 | 2.495128 |
| SESN2 | -1.213780774 | 0.431137281 | 1.304856206 | 2.470591018 |
| TNPO1 | -0.774767066 | 0.584482986 | 1.303470399 | 2.468218986 |
| NAA15 | -0.719995877 | 0.607099177 | 1.302895125 | 2.46723498 |
| C17orf51 | -0.810317663 | 0.570256281 | 1.285728896 | 2.438051998 |
| ZSCAN32 | -0.818039356 | 0.567212269 | 1.281154364 | 2.430333609 |
| DDHD1 | -0.829065614 | 0.562893692 | 1.276297325 | 2.422165301 |
| RHOBTB3 | -1.455671695 | 0.364585302 | 1.271478873 | 2.414089014 |
| PIGW | -1.71996644 | 0.303555782 | 1.249240545 | 2.377162529 |
| FEM1C | -1.611182818 | 0.327329874 | 1.241845538 | 2.365008778 |
| BLVRA | -0.596374005 | 0.661414232 | 1.238812281 | 2.360041585 |
| MEPCE | -0.855313463 | 0.552745215 | 1.219075344 | 2.327974643 |
| CAPN7 | -0.604616592 | 0.657646132 | 1.214940754 | 2.3213125 |
| FAM20B | -1.914337433 | 0.265293745 | 1.209768292 | 2.313004851 |
| CERS6 | -2.339420446 | 0.197589687 | 1.209128525 | 2.31197937 |
| IER2 | -3.425985908 | 0.093041237 | 1.20340619 | 2.302827252 |
| NFAT5 | -0.901647879 | 0.535274979 | 1.200793415 | 2.298660518 |
| CCDC137 | -0.914060818 | 0.530689232 | 1.197345779 | 2.293173924 |
| NEMP1 | -1.065491917 | 0.477809712 | 1.193068224 | 2.28638479 |
| ZNF107 | -1.779002233 | 0.291384849 | 1.191629357 | 2.284105609 |
| CASP7 | -0.642704081 | 0.640511296 | 1.182502196 | 2.269700906 |
| GINS1 | -1.817026372 | 0.283805338 | 1.17466628 | 2.257406572 |
| ZZZ3 | -0.587866759 | 0.665325964 | 1.147571902 | 2.215407209 |
| SMAD3 | -1.817003565 | 0.283809824 | 1.138259468 | 2.20115306 |
| KPNA4 | -1.262682823 | 0.41676822 | 1.113857866 | 2.164236059 |
| ZNF12 | -1.084503527 | 0.471554516 | 1.103117479 | 2.148183864 |
| CBX5 | -0.935165859 | 0.522982342 | 1.096366806 | 2.13815554 |
| SLC7A5 | -2.701631432 | 0.153719124 | 1.093677071 | 2.134172916 |
| CYP24A1 | -1.114213913 | 0.461942787 | 1.088664702 | 2.126771004 |
| ABHD15 | -1.623001485 | 0.324659316 | 1.088189889 | 2.126071166 |
| PTPN3 | -0.616469351 | 0.652265238 | 1.076928747 | 2.109540443 |
| C6orf47 | -3.089762869 | 0.117459648 | 1.073346459 | 2.104308842 |
| PLEKHF1 | -1.265540001 | 0.41594365 | 1.052240384 | 2.073747704 |
| PLAU | -0.881086317 | 0.542958442 | 1.047060089 | 2.066314837 |
| BRI3BP | -3.757889066 | 0.073920121 | 1.046754713 | 2.065877505 |
| MARVELD3 | -0.905731141 | 0.533762131 | 1.042561141 | 2.059881209 |
| TMEM185B | -1.776842627 | 0.291821356 | 1.02230569 | 2.031162535 |
| MIEF2 | -1.22116565 | 0.428936012 | 1.012565692 | 2.017495831 |
| SUV39H1 | -1.026826452 | 0.490788568 | 1.010627066 | 2.014786635 |
| GNA13 | -0.668417718 | 0.629196383 | 0.983806053 | 1.977675948 |
| ZNF558 | -0.595640596 | 0.661750555 | 0.983659568 | 1.977475154 |
| ICOSLG | -1.034172396 | 0.488295912 | 0.973597053 | 1.963730641 |
| SAMD8 | -1.135445602 | 0.4551943 | 0.97212391 | 1.961726489 |
| MORC3 | -0.616120077 | 0.65242317 | 0.958975202 | 1.943928562 |
| LRRC8B | -1.074261426 | 0.474914126 | 0.958125215 | 1.942783603 |
| GJB5 | -1.678398278 | 0.312429313 | 0.950942086 | 1.933134591 |
| CDC40 | -0.593240344 | 0.662852444 | 0.937711509 | 1.915487365 |
| ZBTB39 | -0.99681173 | 0.501106192 | 0.937050611 | 1.914610082 |
| THBS1 | -0.648605765 | 0.637896485 | 0.930211212 | 1.905554951 |
| CWC22 | -0.617319981 | 0.651880768 | 0.921216217 | 1.893711052 |
| FER | -0.622563349 | 0.649515856 | 0.91515935 | 1.88577735 |
| UBQLN2 | -3.253958265 | 0.104824055 | 0.909511647 | 1.878409548 |
| BAG4 | -1.442365715 | 0.367963427 | 0.90093025 | 1.867269611 |
| TIAF1 | -0.886190355 | 0.54104093 | 0.890073859 | 1.853271 |
| ZBTB7B | -0.606089524 | 0.656975045 | 0.879226141 | 1.839388392 |
| UBASH3B | -0.686266728 | 0.621459927 | 0.877833611 | 1.837613819 |
| FAM167A | -0.912110939 | 0.531406972 | 0.865852777 | 1.82241658 |
| CMTM6 | -2.61192516 | 0.163580745 | 0.843405376 | 1.794280415 |
| GK5 | -1.007531278 | 0.497396659 | 0.842172823 | 1.792748143 |
| UBXN2B | -0.934878256 | 0.523086609 | 0.828336011 | 1.775636181 |
| ASH2L | -0.630590968 | 0.645911778 | 0.823902615 | 1.770188033 |
| MVB12B | -0.995897388 | 0.501423881 | 0.817875043 | 1.762807626 |
| USP37 | -0.751069927 | 0.594162753 | 0.809599978 | 1.75272539 |
| NUFIP2 | -1.669218325 | 0.314423657 | 0.808696301 | 1.751627859 |
| KBTBD7 | -1.896055961 | 0.268676871 | 0.799663066 | 1.740694549 |
| ZFP90 | -0.62046583 | 0.650460867 | 0.792111228 | 1.731606628 |
| STBD1 | -1.512536017 | 0.350494566 | 0.76978477 | 1.7050154 |
| MAGT1 | -1.049691299 | 0.483071519 | 0.768418327 | 1.703401266 |
| MSANTD3-TMEFF1 | -0.85131462 | 0.554279433 | 0.766143532 | 1.700717514 |
| RAPGEF5 | -0.896455799 | 0.537204837 | 0.762828782 | 1.696814414 |
| VGLL4 | -0.729271805 | 0.603208304 | 0.757376847 | 1.690414265 |
| NICN1 | -1.086194996 | 0.471001972 | 0.756703152 | 1.689625077 |
| ZNF792 | -0.653351719 | 0.63580148 | 0.746496429 | 1.677713568 |
| KLF10 | -1.030018699 | 0.489703801 | 0.73880819 | 1.668796676 |
| TEAD1 | -0.841944975 | 0.557890939 | 0.728375899 | 1.656772944 |
| CSRNP1 | -0.92504563 | 0.52666386 | 0.713057691 | 1.639274766 |
| NHLRC2 | -1.635395549 | 0.321882144 | 0.699084338 | 1.623474065 |
| ARL4D | -1.636763275 | 0.321577133 | 0.695548422 | 1.619499948 |
| SPATA5 | -0.671918086 | 0.627671633 | 0.689559257 | 1.612790736 |
| TYW3 | -0.94770142 | 0.518457839 | 0.689167586 | 1.612352946 |
| UBQLN4 | -0.945552029 | 0.519230836 | 0.688718517 | 1.611851146 |
| SCN8A | -0.699059262 | 0.615973734 | 0.687068523 | 1.610008744 |
| ADIPOR2 | -0.700721961 | 0.615264236 | 0.684877633 | 1.607565624 |
| ZWINT | -0.666212938 | 0.630158679 | 0.671917531 | 1.593189118 |
| INTS5 | -3.369923777 | 0.096727922 | 0.668165268 | 1.589050824 |
| PREPL | -0.59166276 | 0.663577668 | 0.664392681 | 1.58490095 |
| LYSMD3 | -1.796751134 | 0.287822018 | 0.658056705 | 1.577955702 |
| TPGS1 | -1.198689934 | 0.435670721 | 0.647454782 | 1.566402293 |
| SAPCD1 | -0.594492112 | 0.662277563 | 0.640241305 | 1.558589827 |
| ZNF273 | -0.601182117 | 0.659213587 | 0.624485108 | 1.541660515 |
| SDC1 | -1.279813403 | 0.411848773 | 0.619037513 | 1.535850204 |
| GPRASP2 | -0.694368177 | 0.617979901 | 0.617445145 | 1.534155952 |
| MBNL2 | -0.805486934 | 0.572168931 | 0.615475732 | 1.532063115 |
| MXRA7 | -1.187328076 | 0.439115366 | 0.614139562 | 1.530644832 |
| SERINC3 | -0.976666716 | 0.508152449 | 0.60006841 | 1.515788441 |
| AMMECR1 | -1.92580552 | 0.263193266 | 0.598937703 | 1.514600912 |
| FBXO5 | -0.808778513 | 0.570864988 | 0.598826918 | 1.51448461 |
| RNGTT | -0.909755425 | 0.532275318 | 0.591919812 | 1.507251131 |
