# Supplementary material for: LAST, a c-Myc-inducible long noncoding RNA, cooperates with CNBP to promote CCND1 mRNA stability in human cells
Source: eLife. 2017 Dec 4;6:e30433. doi: 10.7554/eLife.30433 (PMC5739540; doi:10.7554/eLife.30433)
Supplement: Supplementary file 3. [file elife-30433-supp3.doc]

**Supplementary file 3. *LAST* knockdown mRNA sequencing dataset (downregulation).**

**#log2(fold change) <= - 0.58**

**#p-value cut-off <= 1.00**

**#q-value cut-off <= 1.00**

**#Gene biotype: protein coding**

**#Condition pairs: down sh-LAST-1 vs sh-ctrl**

**^a^ Log2(fold change), if the comparison is test vs control, log2 of the fold change will be calculated by log2(Test FPKM+1)-log2(Control FPKM+1).**

**^b^ Fold Change, 2^(log2(fold change)).**

| **Gene Name** | **log2(fold change)^a^** | **Fold Change^b^** | **Sh-ctrl FPKM** | **Sh-LAST-1 FPKM** |
| --- | --- | --- | --- | --- |
| TOMM6 | -5.20959 | 0.027024 | 36.00361 | 0 |
| CEBPG | -4.33017 | 0.049715 | 20.43061 | 0.065429 |
| CSNK2A3 | -4.1859 | 0.054944 | 17.20045 | 0 |
| PRNP | -4.15539 | 0.056118 | 44.26897 | 1.540408 |
| CHMP1B | -3.88142 | 0.067854 | 13.73748 | 0 |
| BRI3BP | -3.75789 | 0.07392 | 24.74984 | 0.903431 |
| MSX1 | -3.68345 | 0.077834 | 33.84385 | 1.712046 |
| HS6ST1 | -3.61856 | 0.081415 | 11.86228 | 0.047183 |
| THAP11 | -3.5611 | 0.084723 | 10.80313 | 0 |
| C16orf91 | -3.51608 | 0.087408 | 17.96335 | 0.657556 |
| VMA21 | -3.47638 | 0.089848 | 17.12134 | 0.628159 |
| ATXN7L3B | -3.46608 | 0.090491 | 10.05081 | 0 |
| JUND | -3.4419 | 0.092021 | 21.71221 | 1.089997 |
| IER2 | -3.42599 | 0.093041 | 15.14071 | 0.501752 |
| INTS5 | -3.36992 | 0.096728 | 11.17719 | 0.177874 |
| MRPL57 | -3.33395 | 0.09917 | 22.32006 | 1.312658 |
| UBQLN2 | -3.25396 | 0.104824 | 8.539795 | 0 |
| RBM15B | -3.1623 | 0.1117 | 7.952532 | 0 |
| SOX4 | -3.15751 | 0.112072 | 24.12974 | 1.816332 |
| YOD1 | -3.10654 | 0.116102 | 11.84008 | 0.490757 |
| C6orf47 | -3.08976 | 0.11746 | 7.513562 | 0 |
| TICAM1 | -3.02074 | 0.123216 | 11.57543 | 0.549498 |
| LSM10 | -2.99026 | 0.125847 | 9.698923 | 0.346427 |
| RNF11 | -2.96285 | 0.128261 | 26.87869 | 2.575745 |
| RP11-544M22.13 | -2.78549 | 0.145039 | 9.941787 | 0.586984 |
| TNFRSF6B | -2.75885 | 0.147742 | 10.96814 | 0.768198 |
| CDC42EP4 | -2.73234 | 0.150482 | 8.134236 | 0.374538 |
| ANKRD33B | -2.73117 | 0.150604 | 7.260636 | 0.244083 |
| SLC7A5 | -2.70163 | 0.153719 | 241.2393 | 36.23682 |
| NUDT15 | -2.66893 | 0.157243 | 34.13009 | 4.523952 |
| MZT1 | -2.61796 | 0.162898 | 20.31603 | 2.472347 |
| CMTM6 | -2.61193 | 0.163581 | 32.76965 | 4.524065 |
| PDF | -2.55727 | 0.169897 | 12.91571 | 1.364238 |
| CBX6 | -2.50618 | 0.176021 | 22.88295 | 3.203904 |
| BAG2 | -2.5005 | 0.176716 | 9.395943 | 0.83713 |
| MOCS3 | -2.49072 | 0.177918 | 4.620582 | 0 |
| ATF4 | -2.48683 | 0.178398 | 119.6659 | 20.52651 |
| C5orf51 | -2.44795 | 0.183271 | 9.435667 | 0.912554 |
| AUNIP | -2.43676 | 0.184699 | 7.636482 | 0.595146 |
| MAL2 | -2.42734 | 0.185908 | 92.53195 | 16.38838 |
| HYPK | -2.37181 | 0.193203 | 6.5654 | 0.461655 |
| CERS6 | -2.33942 | 0.19759 | 22.54619 | 3.652484 |
| CDKN1A | -2.29443 | 0.203848 | 40.58865 | 7.477777 |
| TMEM251 | -2.27898 | 0.206043 | 8.048375 | 0.864356 |
| RAB9A | -2.27573 | 0.206508 | 6.562434 | 0.561702 |
| KIAA1143 | -2.1802 | 0.220646 | 13.68761 | 2.240757 |
| NUDT4 | -2.17998 | 0.220679 | 9.548685 | 1.327877 |
| C6orf106 | -2.12439 | 0.229349 | 19.337 | 3.664261 |
| ATP6V0E2 | -2.12252 | 0.229646 | 8.833469 | 1.258213 |
| C1orf74 | -2.11822 | 0.230331 | 3.385334 | 0.010078 |
| RNASEK-C17orf49 | -2.11059 | 0.231552 | 6.948512 | 0.840496 |
| PURB | -2.09466 | 0.234123 | 3.271267 | 0 |
| KRAS | -2.08068 | 0.236403 | 10.8819 | 1.808917 |
| PHF5A | -2.07946 | 0.236603 | 54.80838 | 12.20441 |
| FZD5 | -2.05356 | 0.240888 | 3.187619 | 0.008749 |
| NXPH4 | -2.02966 | 0.244912 | 5.45706 | 0.581413 |
| MRPL14 | -2.01688 | 0.247091 | 49.85174 | 11.56503 |
| C21orf91 | -2.01007 | 0.248262 | 5.870466 | 0.705675 |
| BEX3 | -2.00249 | 0.249569 | 56.37912 | 13.32005 |
| AC007192.4 | -1.98114 | 0.25329 | 4.959569 | 0.5095 |
| TNFAIP8L1 | -1.96576 | 0.256005 | 4.273678 | 0.350088 |
| SPRED1 | -1.95621 | 0.257704 | 8.644154 | 1.485334 |
| SLC1A5 | -1.95301 | 0.258277 | 86.45298 | 21.58708 |
| HOXA11 | -1.94231 | 0.2602 | 2.843196 | 0 |
| NXT2 | -1.93773 | 0.261027 | 5.519076 | 0.701657 |
| AMMECR1 | -1.92581 | 0.263193 | 4.704604 | 0.501413 |
| FAM20B | -1.91434 | 0.265294 | 13.60296 | 2.874073 |
| SNTB2 | -1.90162 | 0.267642 | 8.073859 | 1.428547 |
| KBTBD7 | -1.89606 | 0.268677 | 2.721943 | 0 |
| WDR82 | -1.87911 | 0.271851 | 38.63315 | 9.774322 |
| MSRB1 | -1.87434 | 0.272751 | 8.400303 | 1.563942 |
| TXNDC5 | -1.86799 | 0.273955 | 68.98991 | 18.17408 |
| SIAH2 | -1.84908 | 0.27757 | 16.80052 | 3.940883 |
| BTBD6 | -1.84797 | 0.277783 | 13.75666 | 3.099153 |
| DYNLT3 | -1.83629 | 0.280041 | 10.67869 | 2.270515 |
| GINS1 | -1.81703 | 0.283805 | 13.78553 | 3.196213 |
| SMAD3 | -1.817 | 0.28381 | 17.86697 | 4.354631 |
| TMEM158 | -1.80573 | 0.286035 | 7.012476 | 1.291851 |
| TOMM20 | -1.7992 | 0.287335 | 101.0508 | 28.32273 |
| LYSMD3 | -1.79675 | 0.287822 | 9.680659 | 2.074129 |
| TP53RK | -1.79519 | 0.288134 | 7.533735 | 1.458857 |
| ZNF107 | -1.779 | 0.291385 | 3.123821 | 0.201619 |
| TMEM185B | -1.77684 | 0.291821 | 2.426754 | 0 |
| RHOV | -1.77119 | 0.292966 | 2.64395 | 0.067554 |
| ADNP | -1.74018 | 0.299333 | 21.30453 | 5.676482 |
| PCBD1 | -1.72267 | 0.302986 | 51.97241 | 15.04992 |
| GLRX5 | -1.72177 | 0.303177 | 24.05032 | 6.594689 |
| PIGW | -1.71997 | 0.303556 | 6.983106 | 1.423318 |
| CTD-2207O23.3 | -1.7052 | 0.306679 | 3.504909 | 0.381559 |
| TMEM56 | -1.70309 | 0.307127 | 4.389974 | 0.655406 |
| METTL15 | -1.70168 | 0.307427 | 6.053935 | 1.168573 |
| GPR63 | -1.69767 | 0.308284 | 2.274625 | 0.009515 |
| RUNX3 | -1.68956 | 0.310021 | 5.148851 | 0.906274 |
| SERTAD3 | -1.68673 | 0.31063 | 5.418306 | 0.993717 |
| FSCN1 | -1.68537 | 0.310924 | 63.82194 | 19.15469 |
| GJB5 | -1.6784 | 0.312429 | 2.200724 | 0 |
| NUFIP2 | -1.66922 | 0.314424 | 22.86365 | 6.503295 |
| ZNF264 | -1.65552 | 0.317424 | 3.13039 | 0.311087 |
| CHAC2 | -1.65419 | 0.317715 | 10.47787 | 2.646696 |
| RP4-583P15.15 | -1.65177 | 0.318249 | 2.142199 | 0 |
| CACUL1 | -1.64985 | 0.318672 | 14.1027 | 3.812812 |
| FAM155B | -1.64635 | 0.319447 | 3.326653 | 0.382138 |
| ARL4D | -1.63676 | 0.321577 | 2.316094 | 0.06638 |
| ANKRD40 | -1.6358 | 0.321792 | 35.09474 | 10.615 |
| NHLRC2 | -1.6354 | 0.321882 | 10.29998 | 2.637262 |
| BLCAP | -1.63169 | 0.322711 | 4.448701 | 0.758355 |
| TMEM65 | -1.62318 | 0.324618 | 3.91229 | 0.594619 |
| ABHD15 | -1.623 | 0.324659 | 2.976051 | 0.290862 |
| RP11-1035H13.3 | -1.61766 | 0.325863 | 10.98709 | 2.906145 |
| FEM1C | -1.61118 | 0.32733 | 9.775454 | 2.527128 |
| NHP2 | -1.59157 | 0.331811 | 87.48146 | 28.35912 |
| SSH1 | -1.59046 | 0.332066 | 14.15617 | 4.032841 |
| CDR2 | -1.58487 | 0.333356 | 10.24692 | 2.749223 |
| MRFAP1 | -1.56866 | 0.337122 | 105.3728 | 34.86059 |
| ENDOG | -1.56372 | 0.338277 | 7.41653 | 1.84712 |
| AMOTL2 | -1.56171 | 0.338749 | 19.00758 | 5.777543 |
| ZNF551 | -1.55656 | 0.33996 | 2.88161 | 0.319592 |
| MINPP1 | -1.5562 | 0.340046 | 11.63178 | 3.295392 |
| ANAPC13 | -1.54302 | 0.343167 | 15.19926 | 4.55905 |
| EMC8 | -1.54279 | 0.34322 | 16.62824 | 5.050366 |
| LRRC59 | -1.54147 | 0.343535 | 98.03638 | 33.02243 |
| FAM175B | -1.53583 | 0.344881 | 19.83903 | 6.186983 |
| COX7A2L | -1.52476 | 0.347538 | 11.60407 | 3.380397 |
| KLHL26 | -1.51829 | 0.349099 | 3.45476 | 0.55515 |
| RAB10 | -1.51649 | 0.349535 | 53.46662 | 18.03797 |
| LAGE3 | -1.51605 | 0.349641 | 14.72318 | 4.497473 |
| STOM | -1.51515 | 0.349859 | 8.827053 | 2.438085 |
| HIF1AN | -1.51365 | 0.350224 | 10.7782 | 3.125011 |
| STBD1 | -1.51254 | 0.350495 | 6.216323 | 1.529282 |
| EID1 | -1.50524 | 0.352271 | 22.78966 | 7.380407 |
| TRIM35 | -1.50337 | 0.352729 | 6.810358 | 1.754941 |
| NEU3 | -1.50305 | 0.352807 | 2.891086 | 0.372803 |
| THAP1 | -1.50072 | 0.353378 | 4.732825 | 1.025852 |
| APEX2 | -1.48334 | 0.35766 | 11.92905 | 3.624201 |
| GTF3C6 | -1.47646 | 0.359369 | 30.75123 | 10.41041 |
| GPX1 | -1.46771 | 0.361555 | 28.605 | 9.703834 |
| PPP1R14B | -1.45575 | 0.364566 | 113.0841 | 40.59119 |
| RHOBTB3 | -1.45567 | 0.364585 | 12.62275 | 3.966653 |
| C18orf25 | -1.45281 | 0.365309 | 6.922384 | 1.89412 |
| KHDC1L | -1.45139 | 0.365668 | 3.136661 | 0.512645 |
| SOCS7 | -1.44797 | 0.366536 | 5.370511 | 1.335025 |
| NUDT16 | -1.44771 | 0.366603 | 3.019373 | 0.473514 |
| TGFBR1 | -1.44639 | 0.366939 | 5.361962 | 1.33445 |
| H2AFX | -1.44558 | 0.367144 | 89.66366 | 32.28659 |
| BAG4 | -1.44237 | 0.367963 | 11.59123 | 3.63311 |
| NKX1-2 | -1.43352 | 0.370226 | 3.163238 | 0.541338 |
| C11orf71 | -1.42843 | 0.371535 | 1.691537 | 0 |
| HIP1 | -1.42302 | 0.372931 | 4.686656 | 1.120728 |
| RAB11FIP2 | -1.39603 | 0.379973 | 7.822749 | 2.352404 |
| RAD54L2 | -1.38151 | 0.383818 | 5.534836 | 1.508189 |
| COLGALT1 | -1.38075 | 0.384019 | 29.93599 | 10.88 |
| NPTXR | -1.37518 | 0.385504 | 3.876296 | 0.879834 |
| MRPS34 | -1.35852 | 0.389983 | 78.0142 | 29.81416 |
| CDA | -1.3584 | 0.390014 | 10.46053 | 3.469765 |
| ASNS | -1.35235 | 0.391655 | 49.78192 | 18.88899 |
| CPTP | -1.34335 | 0.394103 | 6.075802 | 1.788596 |
| RP11-315D16.2 | -1.33308 | 0.396921 | 2.062023 | 0.21538 |
| CENPO | -1.32245 | 0.399854 | 9.339602 | 3.134332 |
| FAM217B | -1.31639 | 0.401537 | 4.013614 | 1.013152 |
| C19orf43 | -1.31073 | 0.403117 | 42.0166 | 16.34071 |
| HOXA7 | -1.31039 | 0.403211 | 6.057713 | 1.845751 |
| KRT80 | -1.31005 | 0.403307 | 8.741197 | 2.928694 |
| SSNA1 | -1.29404 | 0.407808 | 35.91676 | 14.05497 |
| SKA1 | -1.29072 | 0.408747 | 7.972309 | 2.667402 |
| FAM104A | -1.28915 | 0.409191 | 15.95893 | 5.939448 |
| CPEB4 | -1.28625 | 0.410016 | 2.498698 | 0.434522 |
| TRAPPC6B | -1.28542 | 0.41025 | 5.558785 | 1.690741 |
| FAM206A | -1.28524 | 0.410302 | 7.819069 | 2.618479 |
| DCBLD2 | -1.28399 | 0.410657 | 77.84601 | 31.37867 |
| CH507-9B2.3 | -1.28303 | 0.410932 | 7.102241 | 2.329469 |
| MUM1L1 | -1.28221 | 0.411166 | 2.864015 | 0.588753 |
| SDC1 | -1.27981 | 0.411849 | 14.55636 | 5.406868 |
| AMOTL1 | -1.27959 | 0.411913 | 8.017531 | 2.714435 |
| CHST7 | -1.26925 | 0.414877 | 1.410354 | 0 |
| KLHL8 | -1.26772 | 0.415315 | 7.354133 | 2.469599 |
| PLEKHF1 | -1.26554 | 0.415944 | 1.589282 | 0.076996 |
| CRLF3 | -1.26526 | 0.416023 | 13.43838 | 5.006703 |
| KPNA4 | -1.26268 | 0.416768 | 14.68558 | 5.53725 |
| ZNF189 | -1.25903 | 0.417824 | 3.230575 | 0.767635 |
| FAM98B | -1.25627 | 0.418625 | 18.33297 | 7.093258 |
| BAK1 | -1.25283 | 0.419624 | 12.25108 | 4.560468 |
| DCK | -1.24996 | 0.42046 | 19.25484 | 7.516342 |
| TMEM128 | -1.24348 | 0.422352 | 12.2139 | 4.580913 |
| RRM2 | -1.24073 | 0.42316 | 91.82947 | 38.28169 |
| ARHGAP11A | -1.24071 | 0.423164 | 32.04594 | 12.98385 |
| MSH6 | -1.22716 | 0.427158 | 9.749654 | 3.591799 |
| MSN | -1.22253 | 0.42853 | 38.09308 | 15.75256 |
| MIEF2 | -1.22117 | 0.428936 | 2.343464 | 0.434132 |
| KCNK1 | -1.21724 | 0.430105 | 8.475503 | 3.07546 |
| SESN2 | -1.21378 | 0.431137 | 6.132967 | 2.075288 |
| CYSRT1 | -1.21087 | 0.432008 | 1.46861 | 0.06646 |
| FCF1 | -1.2037 | 0.43416 | 15.57753 | 6.197303 |
| SGMS1 | -1.20107 | 0.434953 | 4.027684 | 1.186808 |
| TPGS1 | -1.19869 | 0.435671 | 1.961566 | 0.290267 |
| RNF217 | -1.19812 | 0.435844 | 2.88484 | 0.693184 |
| ACADSB | -1.19768 | 0.435975 | 7.743631 | 2.812008 |
| SPRYD7 | -1.19376 | 0.437161 | 8.969023 | 3.358064 |
| ACTR1A | -1.19052 | 0.438145 | 25.21415 | 10.4856 |
| MXRA7 | -1.18733 | 0.439115 | 7.674214 | 2.808981 |
| SBDS | -1.18606 | 0.439502 | 37.89554 | 16.09468 |
| TMEM43 | -1.18333 | 0.440335 | 18.81406 | 7.724824 |
| ZNF552 | -1.17896 | 0.441671 | 3.582241 | 1.023843 |
| MBTPS2 | -1.17033 | 0.444321 | 4.469992 | 1.430433 |
| E2F1 | -1.16851 | 0.44488 | 16.72982 | 6.887643 |
| UNG | -1.16764 | 0.44515 | 42.51505 | 18.3707 |
| DDX21 | -1.16762 | 0.445156 | 143.6553 | 63.39419 |
| EMC7 | -1.16632 | 0.445555 | 25.97234 | 11.01767 |
| MTMR6 | -1.16571 | 0.445746 | 9.307505 | 3.594524 |
| GNS | -1.16435 | 0.446164 | 13.00781 | 5.249786 |
| SUDS3 | -1.16433 | 0.44617 | 12.97038 | 5.233164 |
| GXYLT1 | -1.16194 | 0.446911 | 2.765775 | 0.682965 |
| SRSF3 | -1.16119 | 0.447145 | 53.31148 | 23.28509 |
| DDIT3 | -1.16096 | 0.447215 | 5.513913 | 1.913118 |
| ZNF217 | -1.15689 | 0.448477 | 12.15852 | 4.90129 |
| ZNF469 | -1.15513 | 0.449024 | 1.227051 | 0 |
| TAGLN2 | -1.15383 | 0.449429 | 38.76563 | 16.87185 |
| PRKX | -1.15263 | 0.449804 | 5.213266 | 1.794755 |
| VTI1B | -1.1523 | 0.449907 | 9.547724 | 3.745494 |
| LMAN2L | -1.15144 | 0.450175 | 7.040436 | 2.619606 |
| GTF2F2 | -1.15057 | 0.450448 | 24.81778 | 10.62957 |
| ZNF572 | -1.1494 | 0.450812 | 1.394207 | 0.079338 |
| ATP1B1 | -1.14711 | 0.45153 | 17.28873 | 7.257914 |
| TMEM41B | -1.14637 | 0.45176 | 11.2481 | 4.5332 |
| FAM174A | -1.14227 | 0.453048 | 5.754373 | 2.060052 |
| ZNF251 | -1.14106 | 0.453425 | 3.290862 | 0.945586 |
| HMG20A | -1.13854 | 0.45422 | 5.980341 | 2.17061 |
| SAMD8 | -1.13545 | 0.455194 | 5.136042 | 1.793091 |
| MESDC2 | -1.12977 | 0.456987 | 6.109938 | 2.249149 |
| RPS10-NUDT3 | -1.12367 | 0.458926 | 1.179 | 0 |
| ZNF618 | -1.12345 | 0.458995 | 1.764563 | 0.268921 |
| DUSP3 | -1.1221 | 0.459426 | 12.41637 | 5.16383 |
| RP11-468E2.4 | -1.11898 | 0.460421 | 3.081622 | 0.879263 |
| CYP24A1 | -1.11421 | 0.461943 | 11.55044 | 4.797586 |
| TRIB3 | -1.1086 | 0.463745 | 15.32059 | 6.568595 |
| ARPC5L | -1.10802 | 0.46393 | 16.76873 | 7.243456 |
| SCAMP4 | -1.10701 | 0.464256 | 7.270911 | 2.839823 |
| C8orf37 | -1.10311 | 0.465513 | 3.382645 | 1.040178 |
| HIST2H2AA3 | -1.10179 | 0.465938 | 1.14621 | 0 |
| C17orf89 | -1.09931 | 0.466739 | 38.63614 | 17.49974 |
| AGK | -1.09753 | 0.467316 | 4.971535 | 1.790591 |
| FAM25A | -1.08926 | 0.470002 | 2.230696 | 0.518434 |
| MAT2B | -1.08777 | 0.470489 | 12.66481 | 5.429141 |
| NICN1 | -1.08619 | 0.471002 | 1.2121 | 0.041904 |
| HOXA9 | -1.08505 | 0.471375 | 8.717667 | 3.580665 |
| ZNF12 | -1.0845 | 0.471555 | 8.579181 | 3.517106 |
| UPP1 | -1.0814 | 0.472569 | 4.624517 | 1.657973 |
| LRRC8B | -1.07426 | 0.474914 | 7.536527 | 3.054117 |
| ZNF57 | -1.07364 | 0.475119 | 3.706805 | 1.236291 |
| HSDL2 | -1.07335 | 0.475214 | 16.26303 | 7.203629 |
| CD83 | -1.07195 | 0.475676 | 5.021269 | 1.864171 |
| CYR61 | -1.06929 | 0.476552 | 16.7148 | 7.442028 |
| TMEM192 | -1.0678 | 0.477045 | 2.136004 | 0.496015 |
| ANP32B | -1.06716 | 0.477258 | 133.722 | 63.29712 |
| NEMP1 | -1.06549 | 0.47781 | 10.91157 | 4.691463 |
| DSN1 | -1.06525 | 0.47789 | 16.59969 | 7.410714 |
| ISCA2 | -1.06503 | 0.477962 | 6.02997 | 2.360059 |
| TAOK1 | -1.06395 | 0.478321 | 11.58285 | 5.018643 |
| RIDA | -1.06081 | 0.479364 | 20.94159 | 9.518004 |
| DPH3 | -1.05933 | 0.479856 | 4.595665 | 1.685113 |
| MOB4 | -1.05797 | 0.480309 | 11.47221 | 4.990514 |
| PLA2G12A | -1.05466 | 0.481411 | 8.273236 | 3.464241 |
| BTBD2 | -1.05361 | 0.481762 | 8.113855 | 3.390706 |
| MAGT1 | -1.04969 | 0.483072 | 12.35191 | 5.449926 |
| RAC1 | -1.04366 | 0.485095 | 32.32631 | 15.16642 |
| LRRC3 | -1.04101 | 0.485987 | 1.131487 | 0.035874 |
| ATP13A3 | -1.03754 | 0.487159 | 31.98077 | 15.06687 |
| DFFA | -1.0361 | 0.487645 | 16.85843 | 7.708566 |
| ICOSLG | -1.03417 | 0.488296 | 1.823493 | 0.3787 |
| KLF10 | -1.03002 | 0.489704 | 30.30301 | 14.3292 |
| N6AMT1 | -1.02925 | 0.489964 | 3.571716 | 1.239977 |
| SUV39H1 | -1.02683 | 0.490789 | 6.745474 | 2.80139 |
| CTD-2410N18.5 | -1.02501 | 0.491407 | 1.034972 | 0 |
| LY6E | -1.02045 | 0.492964 | 38.3425 | 18.39444 |
| ZNF597 | -1.01511 | 0.494791 | 1.142913 | 0.060295 |
| ERLIN2 | -1.01369 | 0.495277 | 5.549086 | 2.243612 |
| ZCCHC17 | -1.0134 | 0.495377 | 16.6631 | 7.749901 |
| SAMD5 | -1.01313 | 0.495472 | 2.29456 | 0.632361 |
| VAMP3 | -1.01208 | 0.495831 | 32.88773 | 15.80258 |
| FAM111A | -1.00927 | 0.496796 | 8.038668 | 3.490373 |
| SLC7A11 | -1.00853 | 0.497054 | 17.97637 | 8.432274 |
| ACBD3 | -1.00831 | 0.497129 | 16.23535 | 7.5682 |
| GK5 | -1.00753 | 0.497397 | 2.283902 | 0.633402 |
| UBE2B | -1.00625 | 0.497838 | 14.09671 | 6.515715 |
| EXOC8 | -1.00585 | 0.497976 | 1.00813 | 0 |
| RAD21 | -1.00506 | 0.498249 | 76.25477 | 37.4921 |
| GSTCD | -1.0018 | 0.499377 | 5.964749 | 2.478034 |
| ZNHIT6 | -0.9979 | 0.500727 | 4.487598 | 1.747788 |
| ZBTB39 | -0.99681 | 0.501106 | 0.995585 | 0 |
| MVB12B | -0.9959 | 0.501424 | 1.551684 | 0.279475 |
| ADIPOR1 | -0.99457 | 0.501887 | 33.31501 | 16.22226 |
| FKBP9 | -0.9934 | 0.502293 | 12.45128 | 5.756478 |
| GPD2 | -0.99258 | 0.502579 | 14.37629 | 6.727799 |
| RABL3 | -0.9918 | 0.502849 | 6.741713 | 2.892915 |
| HIGD2A | -0.9879 | 0.504211 | 71.33765 | 35.47343 |
| MEST | -0.98415 | 0.505525 | 8.494227 | 3.79957 |
| GPN1 | -0.98261 | 0.506064 | 13.18795 | 6.180017 |
| NFE2L1 | -0.98043 | 0.506829 | 25.4701 | 12.41582 |
| ZNF784 | -0.97818 | 0.50762 | 1.207395 | 0.120518 |
| C22orf29 | -0.97761 | 0.50782 | 6.020726 | 2.565268 |
| SERINC3 | -0.97667 | 0.508152 | 22.09978 | 10.73821 |
| EGR1 | -0.97585 | 0.50844 | 0.966801 | 0 |
| PHGDH | -0.97494 | 0.508762 | 13.61313 | 6.434605 |
| HSPE1-MOB4 | -0.97381 | 0.509158 | 7.077929 | 3.112942 |
| TERF1 | -0.97278 | 0.509525 | 5.198402 | 2.158238 |
| MALT1 | -0.97176 | 0.509885 | 15.22492 | 7.272845 |
| CENPM | -0.96883 | 0.51092 | 9.606996 | 4.419328 |
| SMAD4 | -0.96747 | 0.511404 | 5.956313 | 2.557484 |
| TNFRSF10B | -0.96561 | 0.512063 | 14.70049 | 7.039648 |
| C5orf22 | -0.96287 | 0.513036 | 9.858329 | 4.570716 |
| BPGM | -0.96149 | 0.513525 | 3.846653 | 1.488879 |
| GRPEL1 | -0.95562 | 0.515618 | 22.39458 | 11.06268 |
| RPUSD4 | -0.95009 | 0.517599 | 11.4153 | 5.42615 |
| MRPS2 | -0.94967 | 0.517752 | 20.81376 | 10.29411 |
| SNAPC2 | -0.94838 | 0.518215 | 9.882643 | 4.639545 |
| TYW3 | -0.9477 | 0.518458 | 17.36341 | 8.520655 |
| ONECUT3 | -0.94749 | 0.518535 | 5.102038 | 2.164121 |
| SMDT1 | -0.94682 | 0.518775 | 8.148995 | 3.746271 |
| UBQLN4 | -0.94555 | 0.519231 | 27.89476 | 14.00305 |
| CDT1 | -0.9453 | 0.519323 | 36.29832 | 18.36988 |
| R3HDM4 | -0.94493 | 0.519454 | 17.72745 | 8.728049 |
| RP11-371E8.4 | -0.94469 | 0.519541 | 0.924775 | 0 |
| CMTM3 | -0.94331 | 0.520039 | 3.575109 | 1.379237 |
| BRF2 | -0.94245 | 0.52035 | 4.925503 | 2.083335 |
| VSIG10 | -0.94032 | 0.521118 | 4.736135 | 1.989203 |
| CBX5 | -0.93517 | 0.522982 | 11.21591 | 5.388707 |
| UBXN2B | -0.93488 | 0.523087 | 8.010304 | 3.713169 |
| COMMD2 | -0.93125 | 0.524405 | 5.802089 | 2.567048 |
| PHIP | -0.93055 | 0.524659 | 8.127872 | 3.789018 |
| C11orf57 | -0.93018 | 0.524794 | 5.948885 | 2.646732 |
| ID1 | -0.92583 | 0.526377 | 128.1328 | 66.97256 |
| CSRNP1 | -0.92505 | 0.526664 | 2.991409 | 1.102131 |
| OSBPL11 | -0.92476 | 0.52677 | 7.719607 | 3.593225 |
| RBMX2 | -0.92297 | 0.527421 | 11.33951 | 5.508117 |
| ORC6 | -0.92287 | 0.527459 | 10.74464 | 5.194821 |
| CASP2 | -0.92095 | 0.52816 | 9.678097 | 4.639739 |
| MRTO4 | -0.9209 | 0.528181 | 51.08133 | 26.50836 |
| CCDC43 | -0.92021 | 0.528433 | 8.260156 | 3.893377 |
| TMEM126A | -0.91999 | 0.528514 | 21.43968 | 10.85969 |
| RNF5 | -0.91782 | 0.529308 | 25.52009 | 13.0373 |
| SH3BP4 | -0.91685 | 0.529666 | 12.61817 | 6.213082 |
| CCDC137 | -0.91406 | 0.530689 | 26.28896 | 13.48196 |
| FAM167A | -0.91211 | 0.531407 | 0.894577 | 0.006791 |
| ZNF574 | -0.91073 | 0.531916 | 0.887113 | 0.003786 |
| RNGTT | -0.90976 | 0.532275 | 11.62106 | 5.717876 |
| RB1 | -0.90915 | 0.532499 | 13.08962 | 6.502703 |
| TFRC | -0.9087 | 0.532665 | 62.0131 | 32.56485 |
| MARVELD3 | -0.90573 | 0.533762 | 2.369017 | 0.798253 |
| MGAT1 | -0.90561 | 0.533805 | 2.944085 | 1.105373 |
| METTL14 | -0.90426 | 0.534305 | 6.606882 | 3.064394 |
| MRPS36 | -0.9039 | 0.534439 | 19.07351 | 9.728072 |
| NFAT5 | -0.90165 | 0.535275 | 18.09774 | 9.222544 |
| ZNF180 | -0.89993 | 0.535911 | 1.675537 | 0.43385 |
| TUBE1 | -0.89909 | 0.536225 | 4.734312 | 2.074884 |
| RAPGEF5 | -0.89646 | 0.537205 | 2.423671 | 0.839212 |
| MIEN1 | -0.8962 | 0.537298 | 13.82979 | 6.968024 |
| C15orf52 | -0.89339 | 0.538349 | 2.671301 | 0.976441 |
| MAPKAPK3 | -0.89275 | 0.538587 | 27.50954 | 14.35488 |
| IDI1 | -0.89202 | 0.538859 | 18.8442 | 9.693221 |
| PCGF2 | -0.89185 | 0.538924 | 7.728185 | 3.703825 |
| SNAPIN | -0.89112 | 0.539195 | 12.55097 | 6.306611 |
| SNX24 | -0.88966 | 0.539743 | 2.981434 | 1.148951 |
| GOT1 | -0.88788 | 0.540406 | 35.51025 | 18.73035 |
| FUNDC1 | -0.88781 | 0.540435 | 13.65426 | 6.919676 |
| PAQR5 | -0.88708 | 0.540706 | 9.443582 | 4.646906 |
| TIAF1 | -0.88619 | 0.541041 | 0.848289 | 0 |
| FBXO10 | -0.88584 | 0.541171 | 0.965037 | 0.063421 |
| FUCA1 | -0.88535 | 0.541356 | 8.893594 | 4.35596 |
| CCNG1 | -0.88431 | 0.541748 | 29.37872 | 15.45761 |
| GAREM2 | -0.88352 | 0.542044 | 2.785432 | 1.051871 |
| MFSD14C | -0.88231 | 0.542498 | 5.256711 | 2.394252 |
| PEX14 | -0.88142 | 0.542832 | 7.452164 | 3.588103 |
| PLAU | -0.88109 | 0.542958 | 13.48875 | 6.866789 |
| STX11 | -0.87611 | 0.544834 | 0.83542 | 0 |
| PTMA | -0.87498 | 0.545263 | 345.6171 | 187.9975 |
| ADAT3 | -0.87462 | 0.545398 | 2.999789 | 1.181478 |
| DDA1 | -0.87252 | 0.546192 | 6.234725 | 2.951551 |
| HDAC1 | -0.8716 | 0.546539 | 59.30401 | 31.95847 |
| MTX2 | -0.8711 | 0.54673 | 22.85728 | 12.04348 |
| VCL | -0.86915 | 0.547469 | 44.94176 | 24.1517 |
| GLOD4 | -0.86253 | 0.549987 | 14.2263 | 7.374271 |
| FAM203B | -0.86072 | 0.550676 | 3.350362 | 1.395641 |
| TEN1 | -0.85657 | 0.552264 | 0.872371 | 0.034043 |
| MEPCE | -0.85531 | 0.552745 | 18.99498 | 10.05213 |
| MSANTD3-TMEFF1 | -0.85131 | 0.554279 | 1.214172 | 0.22727 |
| METTL7B | -0.84975 | 0.554881 | 1.669054 | 0.481007 |
| LRR1 | -0.84627 | 0.55622 | 14.07122 | 7.382918 |
| TEAD1 | -0.84194 | 0.557891 | 7.340556 | 3.653121 |
| SYS1 | -0.83705 | 0.559788 | 2.72017 | 1.082505 |
| EEFSEC | -0.83665 | 0.559942 | 5.043123 | 2.383795 |
| RAB4A | -0.83302 | 0.561351 | 15.01669 | 7.990986 |
| DPH2 | -0.83241 | 0.561588 | 21.4669 | 11.61715 |
| TREX1 | -0.83202 | 0.561743 | 1.398158 | 0.347148 |
| SSR2 | -0.83061 | 0.562293 | 11.82205 | 6.20975 |
| DDHD1 | -0.82907 | 0.562894 | 2.908246 | 1.199927 |
| RAD51AP1 | -0.82752 | 0.563497 | 10.73228 | 5.611101 |
| CUEDC1 | -0.82291 | 0.565299 | 8.170655 | 4.184163 |
| GEN1 | -0.82288 | 0.565312 | 5.723201 | 2.800705 |
| TMEM177 | -0.82194 | 0.565681 | 7.941425 | 4.057995 |
| SLC36A1 | -0.82167 | 0.565787 | 2.513568 | 0.987932 |
| TMBIM1 | -0.82151 | 0.56585 | 14.55535 | 7.801997 |
| ZBTB3 | -0.81869 | 0.566956 | 1.173083 | 0.232042 |
| ST20-MTHFS | -0.81846 | 0.567045 | 0.861361 | 0.055476 |
| IRS1 | -0.81834 | 0.567095 | 5.399226 | 2.628971 |
| REEP5 | -0.81812 | 0.567179 | 21.26091 | 11.62592 |
| ZSCAN32 | -0.81804 | 0.567212 | 4.184163 | 1.940521 |
| B4GALNT1 | -0.81787 | 0.567278 | 5.235916 | 2.537497 |
| DLAT | -0.81779 | 0.567311 | 29.07404 | 16.06132 |
| LRRC8D | -0.81617 | 0.567948 | 1.846212 | 0.616502 |
| TUSC2 | -0.81454 | 0.568588 | 11.75691 | 6.253422 |
| BET1 | -0.81438 | 0.568654 | 3.119682 | 1.342673 |
| SLC25A6 | -0.81308 | 0.569166 | 60.77744 | 34.16161 |
| C2orf47 | -0.81235 | 0.569455 | 12.65935 | 6.778387 |
| C17orf51 | -0.81032 | 0.570256 | 2.565063 | 1.033 |
| FBXO5 | -0.80878 | 0.570865 | 15.37818 | 8.349728 |
| AFAP1 | -0.80867 | 0.570906 | 4.257924 | 2.001782 |
| THAP7 | -0.80719 | 0.571493 | 5.047242 | 2.455954 |
| FPGT | -0.80662 | 0.571721 | 1.6089 | 0.491563 |
| MBNL2 | -0.80549 | 0.572169 | 17.43095 | 9.545618 |
| GNB1 | -0.80519 | 0.572286 | 58.29231 | 32.93218 |
| FNBP1 | -0.80512 | 0.572316 | 5.401619 | 2.663748 |
| HRK | -0.80484 | 0.572426 | 0.850942 | 0.059528 |
| PSIP1 | -0.80475 | 0.572462 | 24.41672 | 13.55011 |
| FANCE | -0.80433 | 0.572626 | 8.416005 | 4.391852 |
| SLC25A6 | -0.80415 | 0.572699 | 53.03658 | 29.94669 |
| RHOBTB2 | -0.80295 | 0.573177 | 1.91001 | 0.667951 |
| ZBED3 | -0.79995 | 0.574368 | 2.448454 | 0.980681 |
| METRN | -0.79946 | 0.574565 | 8.39683 | 4.399093 |
| SCAMP2 | -0.79915 | 0.574686 | 10.88803 | 5.83189 |
| SLC25A5 | -0.79773 | 0.575253 | 243.4241 | 139.6057 |
| GTPBP10 | -0.79508 | 0.57631 | 4.37647 | 2.098515 |
| CDCA2 | -0.79427 | 0.576633 | 13.82775 | 7.550174 |
| ZNF691 | -0.79355 | 0.576923 | 2.642414 | 1.101394 |
| MRPL15 | -0.79251 | 0.577338 | 54.66254 | 31.1361 |
| STOX1 | -0.79134 | 0.577808 | 1.393559 | 0.383016 |
| CAND1 | -0.78865 | 0.578887 | 17.58792 | 9.760302 |
| HECTD3 | -0.78788 | 0.579194 | 7.307985 | 3.811933 |
| IL27RA | -0.78487 | 0.580406 | 10.12905 | 5.459363 |
| DCUN1D4 | -0.78322 | 0.581068 | 7.170097 | 3.747381 |
| DDT | -0.78135 | 0.581824 | 44.44896 | 25.44329 |
| CCND1 | -0.77542 | 0.58422 | 215.9479 | 125.7453 |
| TNPO1 | -0.77477 | 0.584483 | 19.24936 | 10.83541 |
| OGFRL1 | -0.77438 | 0.584641 | 3.277042 | 1.500533 |
| EAPP | -0.77276 | 0.585297 | 10.50649 | 5.734708 |
| YARS | -0.77072 | 0.586126 | 29.36735 | 16.7991 |
| PPP4R3B | -0.77 | 0.586419 | 8.780556 | 4.735505 |
| E2F4 | -0.76846 | 0.587044 | 28.63325 | 16.39602 |
| CSTA | -0.76838 | 0.587078 | 5.305518 | 2.70183 |
| DUSP23 | -0.76745 | 0.587456 | 17.49592 | 9.865531 |
| NUCKS1 | -0.76443 | 0.588685 | 76.60436 | 44.68455 |
| CKAP2L | -0.76336 | 0.589121 | 7.345224 | 3.916346 |
| ACTR3 | -0.76255 | 0.589453 | 22.26252 | 12.71216 |
| ERLIN1 | -0.76025 | 0.590394 | 13.89107 | 7.791594 |
| OAZ2 | -0.75734 | 0.591585 | 8.662619 | 4.716261 |
| TTC26 | -0.75613 | 0.592083 | 3.647068 | 1.751451 |
| ZNF613 | -0.75466 | 0.592684 | 0.819475 | 0.078374 |
| USP37 | -0.75107 | 0.594163 | 4.915489 | 2.514763 |
| MIER1 | -0.74913 | 0.594962 | 5.95515 | 3.13805 |
| TNPO3 | -0.74805 | 0.595407 | 27.19975 | 15.79033 |
| KIDINS220 | -0.74782 | 0.595503 | 6.55283 | 3.497731 |
| AIMP2 | -0.74755 | 0.595615 | 25.79048 | 14.95681 |
| ZFR | -0.74641 | 0.596085 | 28.1493 | 16.37546 |
| RPAP2 | -0.74352 | 0.597281 | 2.80646 | 1.273527 |
| M6PR | -0.74317 | 0.597425 | 36.46563 | 21.38292 |
| CTGF | -0.74229 | 0.597788 | 2.83907 | 1.294949 |
| E2F6 | -0.74167 | 0.598045 | 10.83585 | 6.078376 |
| PSPC1 | -0.74165 | 0.598054 | 14.46429 | 8.248486 |
| ENTPD6 | -0.74161 | 0.598072 | 11.97735 | 6.761393 |
| TSN | -0.74111 | 0.59828 | 17.34551 | 9.975751 |
| SPECC1L | -0.73948 | 0.598956 | 7.784594 | 4.261588 |
| ZBED1 | -0.7383 | 0.599443 | 1.339647 | 0.402486 |
| SLC25A11 | -0.73549 | 0.600615 | 18.0565 | 10.44562 |
| C1orf174 | -0.73524 | 0.60072 | 8.206167 | 4.530329 |
| ARHGDIA | -0.73523 | 0.600721 | 77.84964 | 46.36667 |
| SCOC | -0.73508 | 0.600784 | 5.893453 | 3.141473 |
| NHEJ1 | -0.7312 | 0.602402 | 4.259387 | 2.168265 |
| BTNL9 | -0.73083 | 0.602559 | 1.157853 | 0.300233 |
| BMP8B | -0.73058 | 0.602663 | 3.070652 | 1.453233 |
| STRN3 | -0.72984 | 0.602969 | 10.89433 | 6.171911 |
| VGLL4 | -0.72927 | 0.603208 | 4.220285 | 2.14892 |
| WBP1 | -0.72898 | 0.603332 | 4.929812 | 2.577648 |
| ZBTB18 | -0.72878 | 0.603412 | 1.18211 | 0.316711 |
| THAP9 | -0.72716 | 0.60409 | 1.541979 | 0.535584 |
| PANO1 | -0.7263 | 0.604452 | 0.65439 | 0 |
| CCL26 | -0.72511 | 0.604953 | 0.827007 | 0.105253 |
| MICB | -0.7242 | 0.605334 | 6.762898 | 3.699147 |
| NAA15 | -0.72 | 0.607099 | 27.58029 | 16.35107 |
| 7-Sep | -0.71718 | 0.608284 | 15.58965 | 9.091215 |
| STK17A | -0.71637 | 0.608629 | 8.720766 | 4.916336 |
| NT5C | -0.71549 | 0.609 | 21.96474 | 12.98553 |
| PPP2R2D | -0.71346 | 0.609856 | 3.84196 | 1.952896 |
| RPF1 | -0.71284 | 0.610117 | 67.56443 | 40.83234 |
| YDJC | -0.71068 | 0.611033 | 20.63515 | 12.21979 |
| PPM1A | -0.70942 | 0.611566 | 3.790096 | 1.92946 |
| RAB5A | -0.70791 | 0.612206 | 14.90227 | 8.735473 |
| LRIF1 | -0.70738 | 0.612433 | 9.022834 | 5.138314 |
| HMMR | -0.70512 | 0.613392 | 23.25278 | 13.87646 |
| ATP23 | -0.70204 | 0.614702 | 3.489662 | 1.759804 |
| TST | -0.70185 | 0.614785 | 7.555759 | 4.259949 |
| ADIPOR2 | -0.70072 | 0.615264 | 10.7658 | 6.239076 |
| CCAR2 | -0.69934 | 0.615854 | 28.36704 | 17.0858 |
| SCN8A | -0.69906 | 0.615974 | 1.112202 | 0.301061 |
| FAM69A | -0.69882 | 0.616077 | 2.703397 | 1.281576 |
| FANCG | -0.69693 | 0.616882 | 12.13417 | 7.102228 |
| ZMYM1 | -0.69581 | 0.617365 | 4.477547 | 2.381644 |
| KRTAP2-3 | -0.69444 | 0.61795 | 2.271194 | 1.021434 |
| GPRASP2 | -0.69437 | 0.61798 | 0.660683 | 0.026269 |
| DENND6A | -0.69054 | 0.61962 | 7.335966 | 4.165134 |
| SSR3 | -0.69039 | 0.619687 | 29.67189 | 18.00697 |
| NDUFB5 | -0.68735 | 0.620992 | 9.917104 | 5.779439 |
| NR2F1 | -0.68727 | 0.621027 | 14.64496 | 8.715943 |
| TLCD1 | -0.68645 | 0.621381 | 10.07424 | 5.881323 |
| UBASH3B | -0.68627 | 0.62146 | 4.110535 | 2.175993 |
| ZNF561 | -0.68178 | 0.623396 | 3.338612 | 1.704675 |
| SLC16A14 | -0.68042 | 0.623982 | 3.32016 | 1.695703 |
| VAMP8 | -0.68006 | 0.624138 | 28.20841 | 17.23009 |
| CNST | -0.67983 | 0.624239 | 4.990717 | 2.739639 |
| SLC5A6 | -0.67832 | 0.624891 | 11.32272 | 6.700354 |
| SRSF1 | -0.67626 | 0.625787 | 118.3294 | 73.67476 |
| ADGRF1 | -0.67568 | 0.626036 | 14.31989 | 8.590807 |
| NSFL1C | -0.67481 | 0.626413 | 12.91857 | 7.718771 |
| SPATA5 | -0.67192 | 0.627672 | 4.371391 | 2.37147 |
| REEP1 | -0.67174 | 0.627751 | 1.89453 | 0.817044 |
| NEPRO | -0.66937 | 0.62878 | 5.768725 | 3.25604 |
| ERVMER34-1 | -0.66884 | 0.62901 | 2.540367 | 1.226927 |
| GNA13 | -0.66842 | 0.629196 | 3.395457 | 1.765605 |
| BRAP | -0.66737 | 0.629652 | 6.98922 | 4.03043 |
| STEAP1 | -0.66692 | 0.629849 | 10.65556 | 6.341235 |
| RNPEP | -0.66651 | 0.630029 | 25.57147 | 15.74079 |
| ZWINT | -0.66621 | 0.630159 | 39.42143 | 24.47191 |
| TACO1 | -0.666 | 0.63025 | 11.68033 | 6.99178 |
| COPS4 | -0.66589 | 0.6303 | 12.2191 | 7.331994 |
| IL1R1 | -0.66551 | 0.630465 | 1.561223 | 0.61476 |
| TRAF3IP1 | -0.66544 | 0.630496 | 5.306705 | 2.976353 |
| CYP1A1 | -0.66528 | 0.630567 | 2.28809 | 1.07336 |
| GAL | -0.66478 | 0.630784 | 33.48347 | 20.7516 |
| RAC2 | -0.66439 | 0.630953 | 11.35779 | 6.79719 |
| CHD6 | -0.66385 | 0.631191 | 2.831415 | 1.418353 |
| NUDT16L1 | -0.66366 | 0.631276 | 5.583664 | 3.156106 |
| SUSD2 | -0.66345 | 0.631365 | 2.293474 | 1.079384 |
| REEP4 | -0.6631 | 0.631522 | 18.48577 | 11.30569 |
| IRAK1BP1 | -0.66276 | 0.63167 | 1.117707 | 0.337693 |
| HIST1H2BL | -0.66266 | 0.631711 | 0.822443 | 0.151257 |
| SNRNP27 | -0.66261 | 0.631736 | 14.00159 | 8.477052 |
| AP3S2 | -0.66075 | 0.632549 | 2.230758 | 1.043613 |
| RNF2 | -0.65691 | 0.634235 | 10.80326 | 6.486036 |
| CPT1B | -0.65547 | 0.634867 | 2.682089 | 1.337637 |
| GMFB | -0.65485 | 0.635141 | 5.102227 | 2.875775 |
| ZNF792 | -0.65335 | 0.635801 | 0.62577 | 0.033667 |
| SNRPC | -0.65183 | 0.636473 | 59.84482 | 37.72607 |
| NIFK | -0.65158 | 0.636583 | 36.84474 | 23.09134 |
| SAR1A | -0.65148 | 0.636628 | 12.10849 | 7.345224 |
| THRAP3 | -0.6512 | 0.636749 | 69.51396 | 43.89973 |
| DDX18 | -0.651 | 0.636838 | 30.50572 | 19.06405 |
| HNRNPA3 | -0.65038 | 0.637114 | 117.1568 | 74.27935 |
| SP6 | -0.65035 | 0.637126 | 0.592446 | 0.014588 |
| SFXN2 | -0.64976 | 0.637385 | 2.626083 | 1.311211 |
| CNOT6L | -0.64883 | 0.637797 | 2.175648 | 1.025417 |
| TOP2A | -0.64864 | 0.637879 | 47.95239 | 30.22572 |
| THBS1 | -0.64861 | 0.637896 | 8.821779 | 5.265279 |
| MRPL11 | -0.64754 | 0.638369 | 25.65805 | 16.01768 |
| ITPRIP | -0.64665 | 0.638763 | 0.578333 | 0.008181 |
| PIGH | -0.64582 | 0.639128 | 3.483582 | 1.865583 |
| PROSER1 | -0.64441 | 0.639757 | 7.718613 | 4.57779 |
| PHF19 | -0.64321 | 0.640287 | 7.801569 | 4.635526 |
| CASP7 | -0.6427 | 0.640511 | 14.18982 | 8.729253 |
| NDUFB8 | -0.64183 | 0.640899 | 61.91858 | 39.32448 |
| RMDN1 | -0.63723 | 0.642948 | 11.75373 | 7.199983 |
| HSPA8 | -0.63549 | 0.643722 | 433.9901 | 279.0128 |
| RMI1 | -0.63435 | 0.644229 | 7.105973 | 4.222102 |
| SETX | -0.63258 | 0.64502 | 18.989 | 11.89331 |
| CDRT4 | -0.63247 | 0.64507 | 2.114705 | 1.009203 |
| TMEM263 | -0.63222 | 0.645184 | 3.064142 | 1.62212 |
| ARL2BP | -0.63219 | 0.645194 | 10.05796 | 6.134536 |
| MARS | -0.63152 | 0.645497 | 33.33425 | 21.16264 |
| AARS | -0.6306 | 0.645909 | 37.98854 | 24.18304 |
| ASH2L | -0.63059 | 0.645912 | 9.136283 | 5.547145 |
| PCK2 | -0.62959 | 0.646361 | 6.948344 | 4.137496 |
| KRT81 | -0.62952 | 0.646391 | 1.353574 | 0.52133 |
| EPHA2 | -0.62868 | 0.646767 | 59.9991 | 38.45219 |
| KHDRBS1 | -0.62855 | 0.646828 | 54.9514 | 35.19091 |
| SH3BP5L | -0.62805 | 0.647052 | 4.357702 | 2.466713 |
| CST3 | -0.62738 | 0.647352 | 11.03041 | 6.787912 |
| KCNJ11 | -0.62648 | 0.647757 | 0.66848 | 0.080769 |
| C7orf26 | -0.62632 | 0.647829 | 6.783266 | 4.042225 |
| PPP2R5D | -0.62592 | 0.648007 | 21.34579 | 13.48022 |
| COMMD7 | -0.62545 | 0.648218 | 4.06666 | 2.2843 |
| RPP38 | -0.62428 | 0.648742 | 7.542946 | 4.542168 |
| FGF2 | -0.62421 | 0.648774 | 2.25414 | 1.111201 |
| ANKRD54 | -0.62363 | 0.649035 | 3.847883 | 2.146446 |
| LYRM5 | -0.62338 | 0.649149 | 1.663125 | 0.728766 |
| FER | -0.62256 | 0.649516 | 3.106776 | 1.667416 |
| WDR34 | -0.6222 | 0.649677 | 37.88804 | 24.26468 |
| CD99 | -0.62214 | 0.649709 | 4.705873 | 2.707155 |
| TSEN15 | -0.62205 | 0.649745 | 10.45423 | 6.442327 |
| CSNK1G1 | -0.62141 | 0.650034 | 5.032072 | 2.921053 |
| FAF1 | -0.62082 | 0.650301 | 8.272824 | 5.030125 |
| ZFP90 | -0.62047 | 0.650461 | 1.433699 | 0.583026 |
| CALB2 | -0.61869 | 0.651263 | 4.051904 | 2.290119 |
| MAK16 | -0.61785 | 0.651643 | 16.08734 | 10.13484 |
| ARNTL2 | -0.61774 | 0.651691 | 4.273413 | 2.436636 |
| CWC22 | -0.61732 | 0.651881 | 13.11134 | 8.198914 |
| MRRF | -0.61718 | 0.651946 | 7.006215 | 4.219617 |
| PSMA2 | -0.61698 | 0.652034 | 29.68437 | 19.00724 |
| PTPN3 | -0.61647 | 0.652265 | 5.620674 | 3.318436 |
| MORC3 | -0.61612 | 0.652423 | 5.80331 | 3.438637 |
| AOC3 | -0.61603 | 0.652466 | 1.328284 | 0.519126 |
| CIAO1 | -0.61566 | 0.652631 | 19.69374 | 12.50537 |
| CCDC34 | -0.61547 | 0.652717 | 6.736139 | 4.049507 |
| PXMP2 | -0.61368 | 0.653529 | 9.451852 | 5.830586 |
| PSME3 | -0.613 | 0.653835 | 26.07588 | 16.70317 |
| C1QTNF6 | -0.61236 | 0.654127 | 1.331072 | 0.524816 |
| SLC48A1 | -0.61213 | 0.654232 | 3.440393 | 1.905048 |
| TBC1D16 | -0.61054 | 0.654953 | 4.954069 | 2.899633 |
| MTMR10 | -0.6101 | 0.65515 | 2.892941 | 1.550461 |
| VAPB | -0.60903 | 0.655639 | 7.719605 | 4.716913 |
| AL669831.1 | -0.60849 | 0.655882 | 0.524664 | 0 |
| C7orf50 | -0.60827 | 0.655985 | 4.512645 | 2.616211 |
| CCT6A | -0.60794 | 0.656133 | 148.9984 | 97.41887 |
| ZFX | -0.60782 | 0.656186 | 3.756761 | 2.12132 |
| PLD6 | -0.60723 | 0.656455 | 4.371083 | 2.525875 |
| TSG101 | -0.6064 | 0.656836 | 15.5361 | 9.861502 |
| TMEM200B | -0.60614 | 0.656952 | 3.614356 | 2.031409 |
| ZBTB7B | -0.60609 | 0.656975 | 8.758789 | 5.411281 |
| BMP2K | -0.60584 | 0.65709 | 3.023841 | 1.644026 |
| TNFSF18 | -0.60527 | 0.657346 | 0.589762 | 0.045024 |
| SYNM | -0.60525 | 0.657358 | 0.853335 | 0.218304 |
| CAPN7 | -0.60462 | 0.657646 | 7.840261 | 4.813763 |
| KDF1 | -0.60434 | 0.657773 | 11.88441 | 7.475008 |
| MAGEA2 | -0.60419 | 0.657842 | 13.88964 | 8.795028 |
| GADD45B | -0.60401 | 0.657921 | 10.30281 | 6.436353 |
| SLC25A38 | -0.60387 | 0.657988 | 16.268 | 10.36214 |
| ARHGAP33 | -0.6023 | 0.658705 | 1.898423 | 0.909206 |
| DHRS4L2 | -0.60142 | 0.659107 | 4.440911 | 2.586144 |
| ZNF273 | -0.60118 | 0.659214 | 0.73923 | 0.146524 |
| SMCO4 | -0.60105 | 0.659273 | 4.150697 | 2.395717 |
| FAM104B | -0.60093 | 0.659329 | 3.861996 | 2.205657 |
| EXT2 | -0.60059 | 0.659486 | 12.67966 | 8.021546 |
| CFAP20 | -0.59962 | 0.659929 | 16.42439 | 10.49886 |
| NOCT | -0.59957 | 0.659952 | 7.842565 | 4.835672 |
| TRIM68 | -0.59956 | 0.659955 | 1.489614 | 0.643032 |
| SLC35F6 | -0.59944 | 0.660012 | 4.005555 | 2.303725 |
| FKRP | -0.59936 | 0.660048 | 1.215557 | 0.462373 |
| MTRNR2L8 | -0.59934 | 0.660056 | 0.515023 | 0 |
| BORA | -0.59816 | 0.660598 | 11.92396 | 7.537537 |
| SFPQ | -0.59736 | 0.660962 | 58.10783 | 38.06802 |
| NCBP2 | -0.5965 | 0.661356 | 17.40303 | 11.17095 |
| BLVRA | -0.59637 | 0.661414 | 11.12558 | 7.020029 |
| GLMP | -0.59579 | 0.661682 | 4.593579 | 2.70117 |
| ZNF558 | -0.59564 | 0.661751 | 2.869521 | 1.560658 |
| SAPCD1 | -0.59449 | 0.662278 | 1.421119 | 0.603453 |
| UBA2 | -0.59445 | 0.662298 | 27.11964 | 17.62359 |
| SYT1 | -0.59416 | 0.66243 | 11.47915 | 7.266567 |
| EVPL | -0.59368 | 0.662652 | 9.249696 | 5.791986 |
| CDC40 | -0.59324 | 0.662852 | 11.3851 | 7.209494 |
| CITED1 | -0.59208 | 0.663385 | 0.58337 | 0.050384 |
| RRM2B | -0.59205 | 0.663398 | 18.1936 | 11.733 |
| PREPL | -0.59166 | 0.663578 | 8.732699 | 5.458402 |
| DLEU1 | -0.59072 | 0.664011 | 0.746839 | 0.159921 |
| BCL2L12 | -0.59053 | 0.6641 | 14.59068 | 9.353775 |
| THOC7 | -0.59009 | 0.664302 | 28.08144 | 18.31885 |
| HIST1H2BD | -0.58978 | 0.664445 | 0.706062 | 0.133584 |
| PGAP1 | -0.58948 | 0.664581 | 1.454503 | 0.631215 |
| NIP7 | -0.58942 | 0.66461 | 11.94234 | 7.60161 |
| HS1BP3 | -0.58905 | 0.664782 | 1.944778 | 0.957634 |
| ZZZ3 | -0.58787 | 0.665326 | 8.07402 | 5.037181 |
| RAB25 | -0.58775 | 0.665382 | 25.85468 | 16.86862 |
| SOX9 | -0.58531 | 0.666508 | 24.59908 | 16.062 |
