# Supplementary material for: LAST, a c-Myc-inducible long noncoding RNA, cooperates with CNBP to promote CCND1 mRNA stability in human cells
Source: eLife. 2017 Dec 4;6:e30433. doi: 10.7554/eLife.30433 (PMC5739540; doi:10.7554/eLife.30433)
Supplement: Supplementary file 4. [file elife-30433-supp4.doc]

**Supplementary file 4. Correlation between *LAST* and *CCND1* expression levels in fifteen TCGA tumor types.**

| **TCGA Tumor Types** | **Pearson Correlation Coefficient (*LAST*-*CCND1*)** | **P value** |
| --- | --- | --- |
| **BLCA** | 0.028 | 0.567 |
| **BRCA** | 0.119 | 0 |
| **CESC** | 0.089 | 0.122 |
| **CHOL** | -0.088 | 0.61 |
| **COAD** | -0.162 | 0.001 |
| **ESCA** | 0.337 | 0 |
| **HNSC** | 0.034 | 0.442 |
| **KIRC** | 0.008 | 0.845 |
| **LIHC** | -0.047 | 0.366 |
| **LUAD** | -0.037 | 0.402 |
| **PAAD** | -0.025 | 0.745 |
| **PRAD** | -0.061 | 0.174 |
| **READ** | -0.277 | 0 |
| **STAD** | 0.167 | 0.001 |
| **UCEC** | 0.024 | 0.58 |
